# Mitochondrial Dysfunction Reveals the Role of mRNA Poly(A) Tail Regulation in Oculopharyngeal Muscular Dystrophy Pathogenesis

**DOI:** 10.1371/journal.pgen.1005092

**Published:** 2015-03-27

**Authors:** Aymeric Chartier, Pierre Klein, Stéphanie Pierson, Nicolas Barbezier, Teresa Gidaro, François Casas, Steven Carberry, Paul Dowling, Laurie Maynadier, Maëlle Bellec, Martine Oloko, Claude Jardel, Bodo Moritz, George Dickson, Vincent Mouly, Kay Ohlendieck, Gillian Butler-Browne, Capucine Trollet, Martine Simonelig

**Affiliations:** 1 mRNA Regulation and Development, Institut de Génétique Humaine, CNRS UPR1142, Montpellier, France; 2 Sorbonne Universités, UPMC Univ Paris 06, UM76, INSERM UMRS974, CNRS FRE3617, Center for Research in Myology, Paris, France; 3 INRA, UMR 866 Différenciation cellulaire et croissance, Montpellier, France; 4 Department of Biology, National University of Ireland, Maynooth, Ireland; 5 Service de Biochimie Métabolique et Centre de Génétique moléculaire et chromosomique, INSERM U1016, Institut Cochin, CNRS UMR 8104, AP-HP, GHU Pitié-Salpêtrière, Paris, France; 6 Institute of Biochemistry and Biotechnology, Martin Luther University Halle-Wittenberg, Halle, Germany; 7 School of Biological Sciences, Royal Holloway - University of London, Egham, Surrey, United Kingdom; Stanford University School of Medicine, UNITED STATES

## Abstract

Oculopharyngeal muscular dystrophy (OPMD), a late-onset disorder characterized by progressive degeneration of specific muscles, results from the extension of a polyalanine tract in poly(A) binding protein nuclear 1 (PABPN1). While the roles of PABPN1 in nuclear polyadenylation and regulation of alternative poly(A) site choice are established, the molecular mechanisms behind OPMD remain undetermined. Here, we show, using *Drosophila* and mouse models, that OPMD pathogenesis depends on affected poly(A) tail lengths of specific mRNAs. We identify a set of mRNAs encoding mitochondrial proteins that are down-regulated starting at the earliest stages of OPMD progression. The down-regulation of these mRNAs correlates with their shortened poly(A) tails and partial rescue of their levels when deadenylation is genetically reduced improves muscle function. Genetic analysis of candidate genes encoding RNA binding proteins using the *Drosophila* OPMD model uncovers a potential role of a number of them. We focus on the deadenylation regulator Smaug and show that it is expressed in adult muscles and specifically binds to the down-regulated mRNAs. In addition, the first step of the cleavage and polyadenylation reaction, mRNA cleavage, is affected in muscles expressing alanine-expanded PABPN1. We propose that impaired cleavage during nuclear cleavage/polyadenylation is an early defect in OPMD. This defect followed by active deadenylation of specific mRNAs, involving Smaug and the CCR4-NOT deadenylation complex, leads to their destabilization and mitochondrial dysfunction. These results broaden our understanding of the role of mRNA regulation in pathologies and might help to understand the molecular mechanisms underlying neurodegenerative disorders that involve mitochondrial dysfunction.

## Author Summary

Oculopharyngeal muscular dystrophy is a genetic disease characterized by progressive degeneration of specific muscles, leading to ptosis (eyelid drooping), dysphagia (swallowing difficulties) and proximal limb weakness. The disease results from mutations in a nuclear protein called poly(A) binding protein nuclear 1 that is involved in polyadenylation of messenger RNAs (mRNAs) and poly(A) site selection. To address the molecular mechanisms involved in the disease, we have used two animal models (*Drosophila* and mouse) that recapitulate the features of this disorder. We show that oculopharyngeal muscular dystrophy pathogenesis depends on defects in poly(A) tail length regulation of specific mRNAs. Because poly(A) tails play an essential role in mRNA stability, these defects result in accelerated decay of these mRNAs. The affected mRNAs encode mitochondrial proteins, and mitochondrial activity is impaired in diseased muscles. These findings have important implications for the development of potential therapies for oculopharyngeal muscular dystrophy, and might be relevant to decipher the molecular mechanisms underlying other disorders that involve mitochondrial dysfunction.

## Introduction

Many neurodegenerative disorders are due to expansions of trinucleotide repeats in the associated genes. In many cases, the pathology is thought to involve protein misfolding and accumulation in insoluble aggregates [1]. However, more recent data have also implicated RNA toxicity and RNA granules in several neurodegenerative diseases [2,3]. RNA repeats can induce the formation of RNA aggregates and interact with RNA binding proteins, thus interfering with RNA metabolism.

Oculopharyngeal muscular dystrophy (OPMD) is another triplet expansion disease which results from short expansions of a GCN repeat in the gene encoding poly(A) binding protein nuclear 1 (PABPN1) [4]. OPMD is an autosomal dominant muscular dystrophy, which has a late onset and is characterised by progressive weakness and degeneration of specific muscles [5,6]. Triplet expansion in *PABPN1* leads to extension of a polyalanine tract from 10 alanines in the normal protein to a maximum of 17 alanines at the N-terminus of the protein. Nuclear aggregates in muscle fibres are a pathological hallmark of OPMD [7]. These aggregates contain mutant insoluble PABPN1, ubiquitin, subunits of the proteasome, as well as poly(A) RNA [8]. Polyalanine expansions in PABPN1 are thought to induce misfolding and formation of aggregates, which are targeted to the ubiquitin-proteasome degradation pathway [9,10]. However, it is still unknown whether these nuclear aggregates have a pathological function, a protective role, or are a consequence of a cellular defence mechanism.

Despite recent progress in OPMD pathophysiology showing important deregulation of the ubiquitin-proteasome system in the disease [9] and a role of apoptosis [11], the molecular mechanisms leading to muscle dysfunction remain undetermined.

PABPN1 plays a role in nuclear polyadenylation, an mRNA processing reaction leading to the formation of the poly(A) tail at the 3' end of mRNAs [12]. PABPN1 binds to nascent poly(A) tails during this reaction and acts in stimulating poly(A) polymerase (the enzyme that synthesizes the poly(A) tail) and controlling poly(A) tail length [13–15]. Consistent with this function, *in vivo* data using mutants of *Pabp2*, the *PABPN1* homologue in *Drosophila* [16], and knock-down of PABPN1 in cultured mouse myoblasts [17] have shown shorter mRNA poly(A) tail lengths upon depletion of PABPN1. Other functions of PABPN1 have been described more recently in i) a nuclear surveillance mechanism leading to hyperadenylation and decay of RNAs retained in the nucleus [18], ii) polyadenylation and turnover of long non-coding RNAs [19], and iii) regulation of poly(A) site usage whereby PABPN1 prevents the utilization of proximal weak poly(A) sites [20]. Any of these functions might be relevant to OPMD, and indeed a general shift in the utilization of proximal poly(A) sites was described in a mouse model of OPMD and correlated with some upregulation of the shorter form of mRNAs generated following this shift [20,21]. However, whether and how this upregulation might underlie OPMD was not addressed.

Here we use a *Drosophila* model of OPMD to investigate the molecular defects leading to OPMD. This model is generated by the expression of alanine expanded mammalian PABPN1 (PABPN1-17ala) specifically in muscles; it reproduces the disease characteristics, namely progressive muscle weakness and degeneration resulting in wing position defects, and formation of nuclear aggregates containing mutant PABPN1 in affected muscles [22,23]. Using complementary transcriptomic and genetic approaches we show that an early defect during OPMD progression is the down-regulation of mRNAs encoding mitochondrial proteins, which depends on their active deadenylation and leads to mitochondrial dysfunction. We find that the cleavage step during the nuclear cleavage/polyadenylation reaction is affected in the *Drosophila* model of OPMD. However, this defect is general and the specific mRNA down-regulation depends on active deadenylation involving the CCR4-NOT deadenylation complex and Smaug (Smg), an RNA binding protein known to recruit the CCR4-NOT complex to specific mRNAs [24,25]. Reducing the dosage of genes involved in deadenylation partially rescues the levels of mRNAs encoding mitochondrial proteins leading to improvement of muscle function. Importantly, these early defects are conserved in a mouse model of OPMD and mitochondrial protein levels are also decreased in muscle biopsies from OPMD patients. Down-regulated mRNAs involved in mitochondrial function are enriched in Smg recognition elements (SREs) in both *Drosophila* and mouse. We propose a model in which the first molecular defect in OPMD concerns PABPN1 nuclear function in cleavage/polyadenylation. Some mRNAs are specifically sensitive to this defect because they are actively deadenylated by Smg and the CCR4-NOT deadenylation complex in the cytoplasm. A subset of these mRNAs encodes mitochondrial proteins and the molecular defect results in a deficit of mitochondrial activity, which in turn affects muscle function.

## Results

### Genes encoding mitochondrial proteins are down-regulated in *Drosophila* muscles expressing PABPN1-17ala

**Fig 1:**
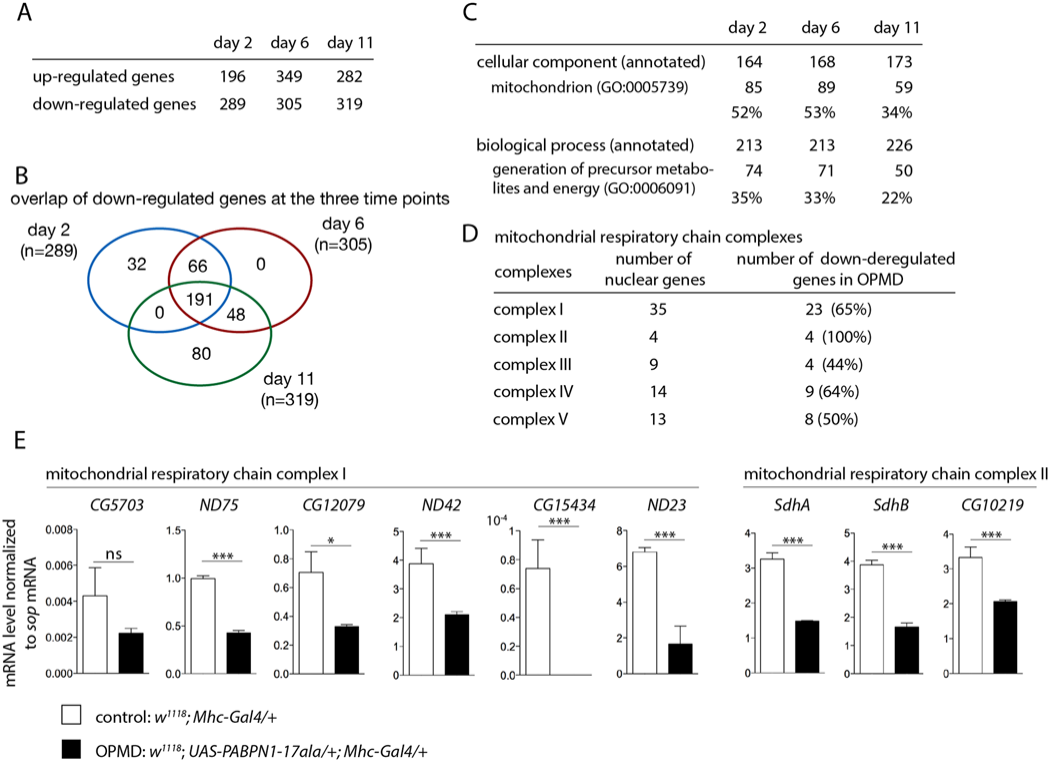
Deregulation of the mitochondrial pathway in the OPMD *Drosophila* model. A) Number of deregulated genes in the transcriptome of PABPN1-17ala-expressing muscles using microarrays. B) Venn’s diagram of overlapping down-regulated genes at days 2, 6 and 11. C) GO term enrichment in down-regulated genes in PABPN1-17ala-expressing muscles. D) Number of nuclear genes encoding mitochondrial respiratory chain complex subunits down-regulated in PABPN1-17ala-expressing muscles. E) Quantification of levels of mRNAs encoding mitochondrial respiratory chain subunits of complexes I and II in control and PABPN1-17ala-expressing muscles at day 2, using RT-qPCR. mRNA levels were normalized to *sop* mRNA. Similar results were obtained when normalization was with *Cpr100A* mRNA which is expressed in thorax cuticle. Means are from three biological replicates, error bars represent standard deviation. * *p*-value <0.05, *** *p*-value <0.001, ns: not significant, using the Student’s t-Test.

To gain insight into the molecular and physiological defects in OPMD we performed a transcriptomic analysis in *Drosophila* muscles expressing PABPN1-17ala. Using microarrays, thorax gene expression was compared between control flies (*Mhc-Gal4/+)* and flies expressing PABPN1-17ala in thoracic muscles (*UAS-PABPN1-17ala/+; Mhc-Gal4/+*), at three time points (days 2, 6 and 11) [26]. We used two-way ANOVA with genotype and time as the first and second variables, respectively, to identify deregulated genes. Up- and down-regulated genes were found at all time points after expression of PABPN1-17ala (Fig. 1A) [26]. We reported the identification of the ubiquitin-proteasome system as a prominent pathway deregulated in OPMD [9]. Importantly, this pathway was identified using the comparison of transcriptomic analyses from the *Drosophila* and mouse models of OPMD and from patient biopsies, demonstrating the relevance of the *Drosophila* and mouse models. In *Drosophila* muscles expressing PABPN1-17ala, genes involved in the ubiquitin-proteasome system were mostly up-regulated at day 6 [9]. Here we focused on deregulated genes at the earliest stage, with the aim of identifying early defects during OPMD progression. At day 2, the most prominent deregulated pathway corresponded to mitochondrial function (see below), and genes involved in this pathway were down-regulated. In total, 289 genes were found to be down-regulated at day 2, among which 191 genes were down-regulated at the three time points (Fig. 1B, S1 Table).

Gene ontology (GO) term enrichment was analysed using FlyMine (http//www.flymine.org) [27] with a *p*-value <0.05 (Bonferroni corrected). Among annotated genes, the term "mitochondrion" in "cellular component" was identified with the strongest *p*-value (1.34E-46 at day 2, 1.85E-41 at day 6 and 2.78E-19 at day 11). Up to 53% of annotated genes were annotated with this term (Fig. 1C). In particular, a large proportion of nuclear genes involved in oxidative phosphorylation (subunits of the respiratory chain complexes) were found to be down-regulated in *Drosophila* muscles expressing PABPN1-17ala (Fig. 1D).

We used RT-qPCR to validate gene down-regulation observed with microarrays. Nineteen genes were analysed, among which 16 (84% validation) were found to be significantly down-regulated in muscles expressing PABPN1-17ala (Fig. 1E, S1A, B Fig). In addition, five additional genes encoding subunits of the respiratory chain complexes (*Ucrh*, *RFeSP*, *Oscp*, *CG1746* and *ATPsyn-b*) were analysed using RT-qPCR and found to be down-regulated (S1A Fig), although they were not detected to be affected using microarrays, indicating the underestimation of the number of deregulated genes. Four other genes (*RpS6*, *RpL32*, *Vha16-1* and *CG1031*), which are not involved in mitochondrial function and were not deregulated in microarrays, served as negative controls and were found to be unaffected using RT-qPCR (S1C Fig).

In summary, the major pathway down-regulated in *Drosophila* muscles expressing PABPN1-17ala corresponded to nuclear genes encoding mitochondrial proteins. This down-regulation started at the earliest time point (day 2), before the onset of muscle degeneration [23], indicating that it is a early defect, that is maintained at later time points.

### Defects in mitochondrial function play an important role in OPMD physiopathology in the *Drosophila* model

**Fig 2:**
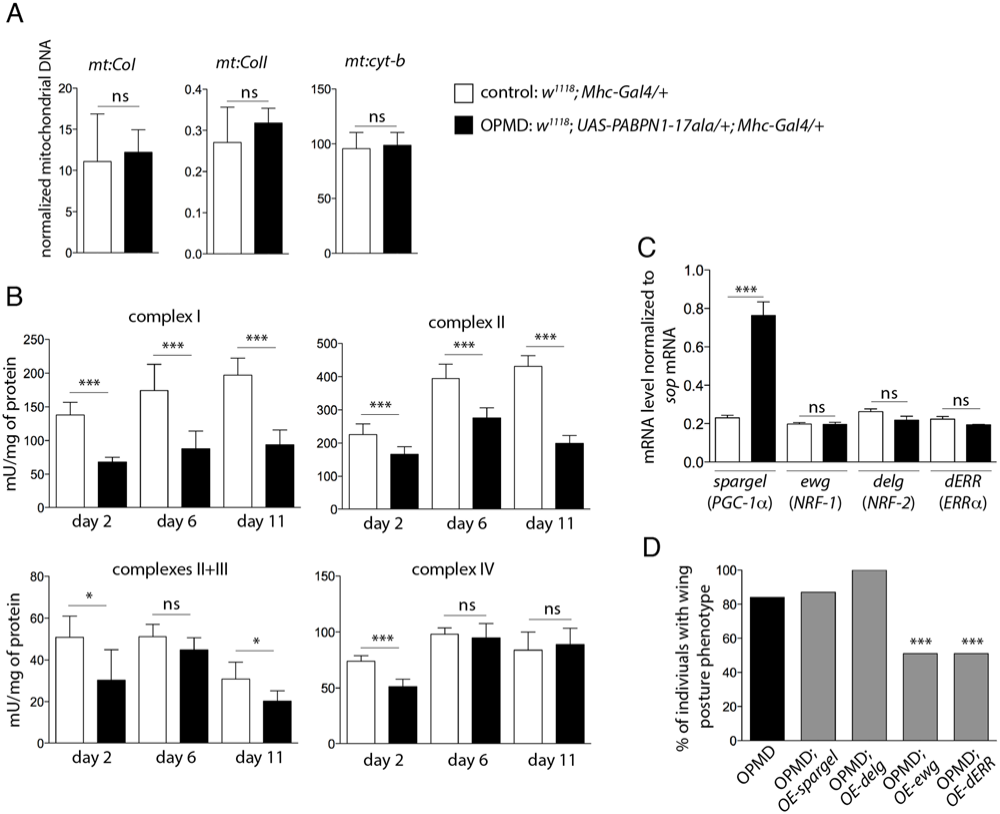
Reduced mitochondrial activity in muscles expressing PABPN1-17ala. A) Quantification of mitochondrial DNA (mtDNA) content in control and PABPN1-17ala- expressing thoraxes at day 2, using qPCR. Three mitochondrial genes (*mt*:*CoI*, *mt*:*CoII* and *mt*:*cyt-b*) were analysed. Mitochondrial DNA levels were normalized to *RpL32* DNA. Means are from three biological replicates, error bars represent standard deviation. ns: not significant, using the Student’s t-Test. B) Activities of mitochondrial respiratory chain complexes were analysed by spectrophotometry from control and PABPN1-17ala-expressing thoraxes (genotypes as in A). Means are from five biological replicates, error bars represent standard deviation. * *p*-value <0.05, *** *p*-value <0.001, ns: not significant, using the Student’s t-Test. C) Quantification of mRNA levels of transcription factors regulating mitochondrial function in control and PABPN1-17ala-expressing thoraxes at day 2, using RT-qPCR (genotypes as in A). mRNA levels were normalized to *sop* mRNA. Means are from two biological replicates quantified three times, error bars represent standard deviation. *** *p*-value <0.001, ns: not significant, using the Student’s t-Test. D) Overexpression of *ewg* and *dERR* genes reduces the wing posture phenotypes of flies expressing PABPN1-17ala. Wing posture phenotypes were scored at day 6, at 18°C from OPMD (*w* ^*1118*^ *; UAS-PABPN1-17ala/+; Mhc-Gal4/+*), OPMD; *OE-spargel* (*w* ^*1118*^ *; UAS-PABPN1-17ala/+; Mhc-Gal4/*s*pargel* ^*EY05931*^), OPMD; *OE-delg* (*w* ^*1118*^ *; UAS-PABPN1-17ala*, *UAS-delg-HA/+; Mhc-Gal4/+*), OPMD; *OE-ewg* (*w*, *ewg* ^*EY05137*^ */Y; UAS-PABPN1-17ala/+; Mhc-Gal4/+*) and OPMD; *OE-dERR* (*w* ^*1118*^ *; UAS-PABPN1-17ala/+; Mhc-Gal4/dERR* ^*G4389*^) flies (n > 130). *** *p*-value <0.001, using the χ^2^ test.

Because down-regulation of mitochondrial components is prominent at the earliest time point, we checked whether mitochondrial biogenesis or mass were affected in muscles expressing PABPN1-17ala. Mitochondrial abundance was analysed by quantifying mitochondrial DNA using qPCR. Mitochondrial DNA levels normalized to nuclear DNA were unaffected in *Drosophila* muscles expressing PABPN1-17ala, suggesting that mitochondrial mass was similar to that in control muscles (Fig. 2A).

Mitochondria generate energy from nutrients through oxidative phosphorylation using the combined action of five enzyme complexes. We assessed the activity of four of these complexes in *Drosophila* muscles, as well as that of citrate synthase which correlates with mitochondrial mass [28]. The activity of citrate synthase was only slightly decreased (83%, 80% and 91% of the control muscle activity at day 2, 6 and 11, respectively), consistent with mitochondrial DNA levels being unaffected in muscles expressing PABPN1-17ala. In contrast, the activities of the four mitochondrial respiratory chain complexes (complex I: NADH dehydrogenase; complex II: succinate dehydrogenase; complex III: cytochrome bc1; and complex IV: cytochrome *c* oxidase) were significantly decreased in muscles expressing PABPN1-17ala (Fig. 2B). These results indicate a mitochondrial dysfunction in the OPMD *Drosophila* model. As another assay to confirm this defect, we used the *GstD1-GFP* transgene as a marker of oxidative stress [29]. Mitochondrial dysfunction leads to oxidative stress [30] which in turn activates the expression of the detoxification enzyme GstD1. Expression of the *GstD1-GFP* transgene was specifically induced in muscles expressing PABPN1-17ala, starting at day 2 (S2 Fig).

Both the generation of oxidative stress and the defect in energy production resulting from decreased mitochondrial respiratory chain activity could participate in the dysfunction of muscles. To functionally address whether mitochondrial activity defects play a role in the dysfunction of muscles expressing PABPN1-17ala, we tested if OPMD phenotypes of defective wing posture could be rescued by increasing the expression of genes involved in mitochondrial function.

Mitochondrial biogenesis and activity are controlled by cellular pathways allowing crosstalk between mitochondria and the cell nucleus. The transcription factors nuclear respiratory factor-1 and -2 (NRF-1 and NRF-2) and oestrogen-related receptor-α (ERRα), as well as the co-activator PPARγ coactivator 1 (PGC-1) are known to co-regulate a large number of genes involved in mitochondrial function, including those encoding subunits of the respiratory chain complexes [31–33]. *NRF-1*, *NRF-2* and *PGC-1* genes are conserved in *Drosophila* (*erect wing* (*ewg*), *delg* and *spargel*, respectively), as well as their function in the control of mitochondrial mass and activity [34–36]. A potential role of *Drosophila* ERR (*dERR*) in mitochondrial function has also been proposed during adult stages [37]. We first checked that the reduced respiratory chain complex activities did not result from decreased expression of these transcription activators in *Drosophila* muscles expressing PABPN1-17ala, using RT-qPCR. The levels of *ewg*, *delg* and *dERR* mRNAs were unaffected in these muscles and *spargel* expression was found to be increased (Fig. 2C). While overexpression of *spargel* or *delg* in muscles was not able to rescue wing posture defects, overexpression of *ewg* or *dERR* significantly reduced these defects (Fig. 2D).

Together, these results show a deficit in mitochondrial respiratory chain activity in *Drosophila* muscles expressing PABPN1-17ala and strongly suggest that this deficit has a key role in muscle defects since overexpression of general regulators of mitochondrial biogenesis and function improved muscle function.

### Role of mRNA poly(A) tail length regulation in the defects induced by PABPN1-17ala expression

**Fig 3:**
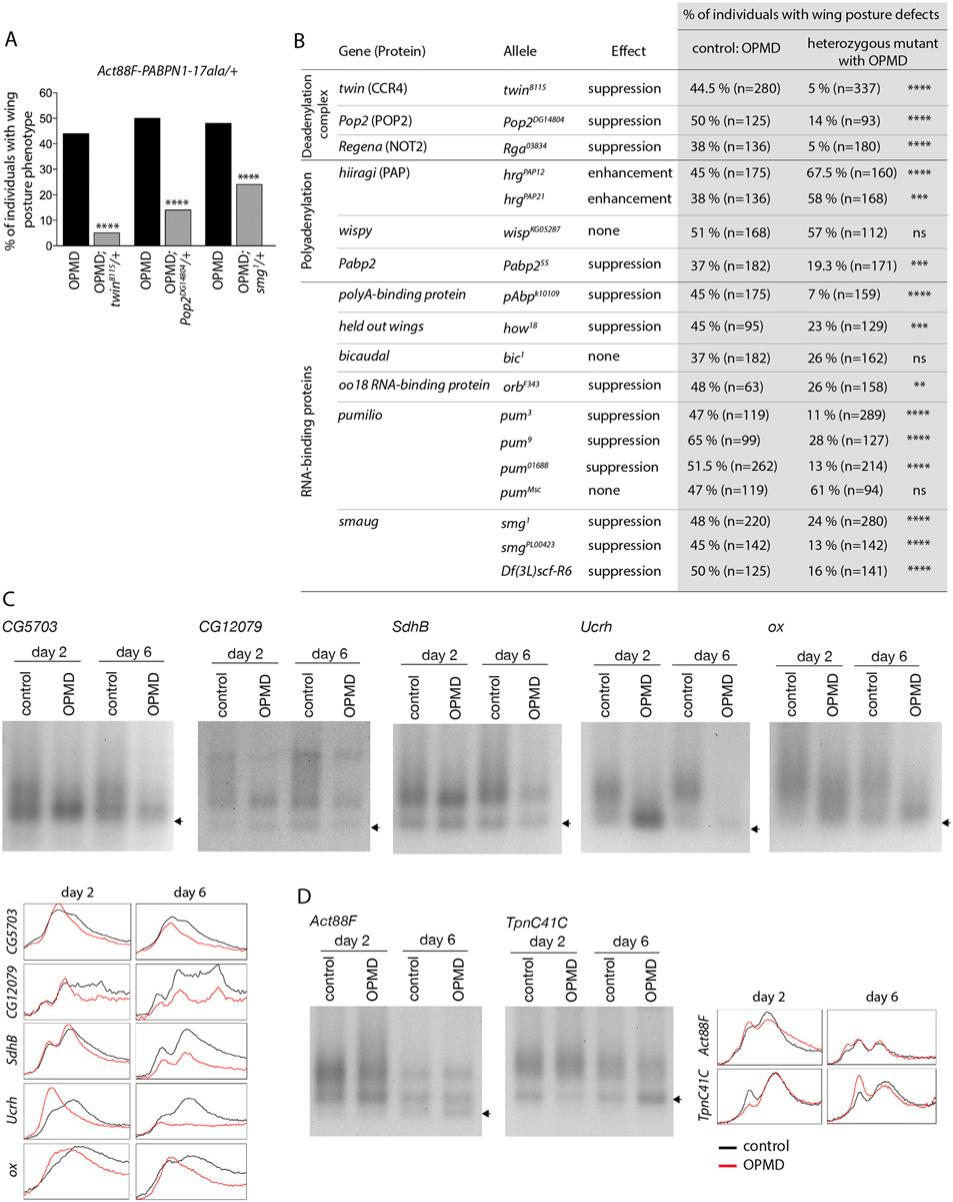
Poly(A) tail length regulation plays a major role in the OPMD *Drosophila* model. A) Genetic rescue of wing position phenotypes with genes involved in poly(A) tail regulation. Percentage of wing posture defects in the presence or absence of heterozygous mutants, scored at day 6. OPMD flies were *w* ^*1118*^ *; Act88F-PABPN1-17ala/+* raised at 25°C. Note that OPMD phenotypes (affected wing posture) are not visible at day 2 with this transgene. **** *p*-value <0.0001, using the χ^2^ test. B) Genetic screen with heterozygous mutants. Wing posture defects were scored at day 6. OPMD flies were *w* ^*1118*^ *; Act88F-PABPN1-17ala/+* raised at 25°C. *wispy* encodes a cytoplasmic poly(A) polymerase expressed in the female germline [42]. **** *p*-value <0.0001, *** *p*-value <0.001, ** *p*-value <0.01, ns: not significant, using the χ^2^ test. C-D) PAT assays in control (*w* ^*1118*^ *; Mhc-Gal4/+*) and OPMD (*w* ^*1118*^ *; UAS-PABPN1-17ala/+; Mhc-Gal4/+*) adult thoraxes from flies raised at 18°C, at days 2 and 6. C) mRNAs encoding subunits of mitochondrial respiratory chain complexes. D) mRNAs encoding muscle sarcomeric proteins. Arrows indicate poly(A) tails of 12A. Profiles of PAT assays using the ImageJ software are shown.

We have shown previously that the RNA binding activity of PABPN1 plays a role in OPMD pathogenesis suggesting that the disease process involves mRNA metabolism [23]. To test this hypothesis, we performed a small screen using heterozygous mutants of genes encoding RNA binding proteins and proteins involved in poly(A) tail length regulation (polyadenylation and deadenylation). We generated a new transgene *Act88F-PABPN1-17ala* which allows constitutive expression of PABPN1-17ala in adult indirect flight muscles [38]. Expression of this transgene led to 40 to 50% of flies showing abnormal wing posture, thus allowing to screen for a decrease or an enhancement of this phenotype (Fig. 3A, B). The CCR4-NOT deadenylation complex is composed of eight subunits in *Drosophila*, including two deadenylases CCR4 and POP2, and is involved in shortening mRNA poly(A) tails [39–41]. Strikingly, we found that all available mutations in the complex subunits decreased the number of *Act88F-PABPN1-17ala/+* flies with wing position defects (Fig. 3A, B). In contrast, mutants of the *hiiragi* gene which encodes the nuclear poly(A) polymerase involved in polyadenylation [42,43] enhanced the wing position defects (Fig. 3B). Reduced dosage of *Pabp2* (*Drosophila PABPN1*) decreased the number of flies with abnormal wing posture, suggesting that part of these phenotypes resulted from a gain-of-function of PABPN1. Several suppressors encoded RNA binding proteins indicating their potential implication in OPMD (Fig. 3B). This is consistent with the important role of mRNA regulation in OPMD and suggests an involvement of multiple RNA-dependent pathways. Among suppressors, *pumilio* (*pum*) and *smg* encode translational repressors that directly interact with the CCR4-NOT deadenylation complex [24,25,44,45].

Deadenylation is the first step of mRNA destabilization, we therefore measured poly(A) tail lengths of a number of mRNAs that were down-regulated in PABPN1-17ala-expressing muscles, namely mRNAs encoding subunits of the mitochondrial respiratory chain complexes, using Poly(A) test assays (PAT assays). These mRNAs had shorter poly(A) tails in muscles expressing PABPN1-17ala starting at day 2 (Fig. 3C, S3 Fig). In contrast, poly(A) tail lengths of control mRNAs encoding myofibril-specific or ribosomal proteins (Actin 88F (Act88F), Troponin C at 41C (TpnC41C) and Sop) were unaffected in muscles expressing PABPN1-17ala, consistent with their unchanged levels in microarrays (Fig. 3D, S3 Fig).

These genetic data indicate that the regulation of mRNA poly(A) tail length plays an essential role in OPMD pathogenesis in the *Drosophila* model; specific mRNAs have shorter poly(A) tail lengths, which leads to their destabilization.

### Affected nuclear cleavage/polyadenylation in the *Drosophila* model of OPMD

**Fig 4:**
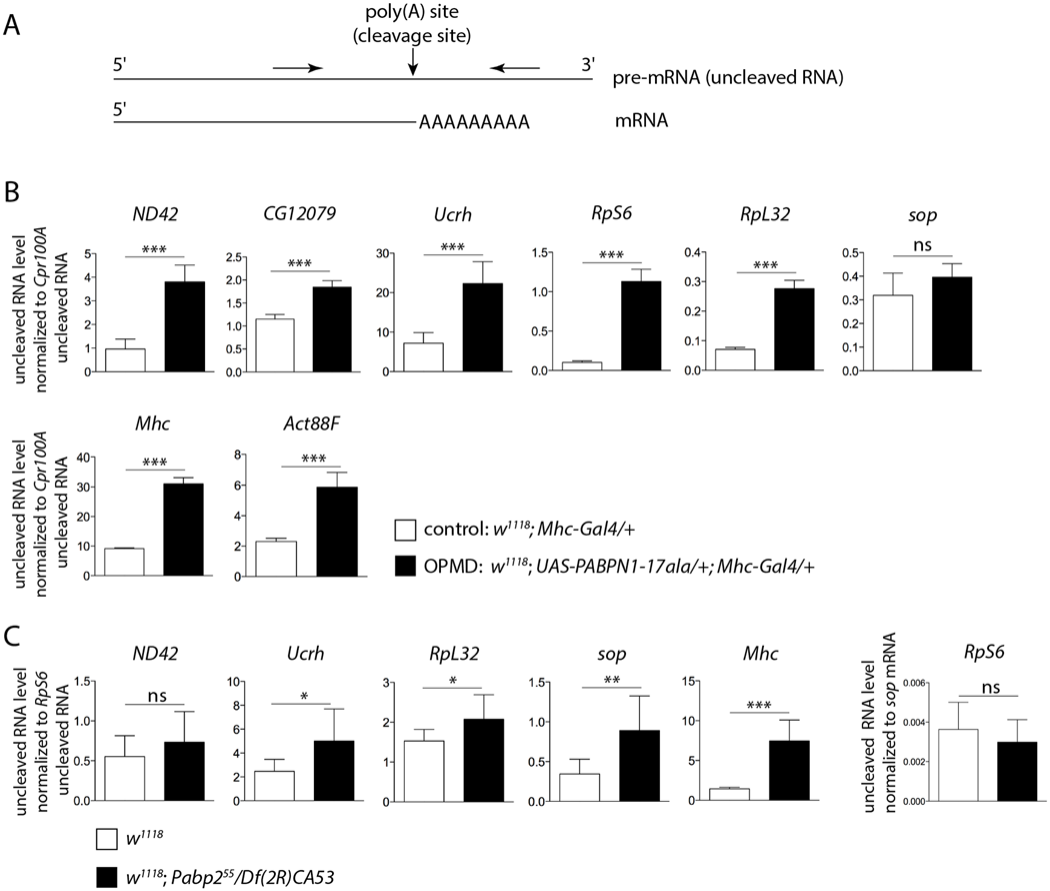
Defective cleavage at poly(A) sites in muscles expressing PABPN1-17ala and in the *Pabp2* mutant. A) Schematic representation of primers (arrows) used to quantify uncleaved pre-mRNA. B) Quantification of uncleaved pre-mRNAs in control and PABPN1-17ala-expressing thoraxes at day 2, using RT-qPCR. Uncleaved RNAs were normalized to *Cpr100A* uncleaved RNA. *Cpr100A* is expressed in the cuticle and its expression remains unaffected by expression of PABPN1-17ala in muscles. Means of three biological replicates quantified three times. For (B) and (C), error bars represent standard deviation. * *p*-value <0.05, ** *p*-value <0.01, *** *p*-value <0.001, ns: not significant, using the Student’s t-Test. C) Quantification of uncleaved pre-mRNAs in control (*w* ^*1118*^) and *Pabp2* (*Pabp2* ^*55*^ */Df(2R)CA53*) mutant first instar larvae, using RT-qPCR. Uncleaved RNAs were normalized to *RpS6* uncleaved RNA. The levels of *RpS6* uncleaved RNA normalized to *sop* mRNA were unaffected in *Pabp2* mutant larvae (right panel). Means of three biological replicates quantified three times.

PABPN1 is mostly nuclear and involved in polyadenylation and in the prevention of weak proximal poly(A) site utilization in cases of several tandem poly(A) sites. This last function indicates a role of PABPN1 during the cleavage step of the cleavage/polyadenylation reaction at weak poly(A) sites, and we asked whether cleavage might be generally impaired in OPMD. We analysed pre-mRNA cleavage at poly(A) sites by quantifying the levels of uncleaved pre-mRNA using RT-qPCR (Fig. 4A). We found that the cleavage at poly(A) sites was less efficient in muscles expressing PABPN1-17ala than in control muscles (Fig. 4B). This defect was not restricted to genes that were down-regulated in muscles expressing PABPN1-17ala (i.e. genes encoding mitochondrial proteins) but also occurred in genes encoding either ribosomal (RpS6, RpL32) or muscle specific (Myosin heavy chain (Mhc), Act88F) proteins, that were not down-regulated.

A general defect in pre-mRNA cleavage during the cleavage/polyadenylation reaction had not been associated previously with *PABPN1* loss-of-function, we therefore tested whether this step of the reaction was affected in *Pabp2* mutants. The null allele *Pabp2* ^*55*^ is lethal at first instar larval stage [16], we used first instar *Pabp2* ^*55*^ */Df(2R)CA53* larvae before they died to quantify the levels of uncleaved pre-mRNA by RT-qPCR. Similarly to what was found in muscles expressing PABPN1-17ala, we observed a decreased efficiency in the cleavage of several pre-mRNAs (Fig. 4C), suggesting that PABP2 plays a general role in the cleavage step of the cleavage/polyadenylation reaction.

These results reveal that in the OPMD *Drosophila* model, the first defect is nuclear and corresponds to a decreased efficiency of cleavage at poly(A) sites. This defect does not systematically lead to reduced steady-state levels of mature mRNAs.

### Smg regulates mRNAs down-regulated in muscles expressing PABPN1-17ala

**Fig 5:**
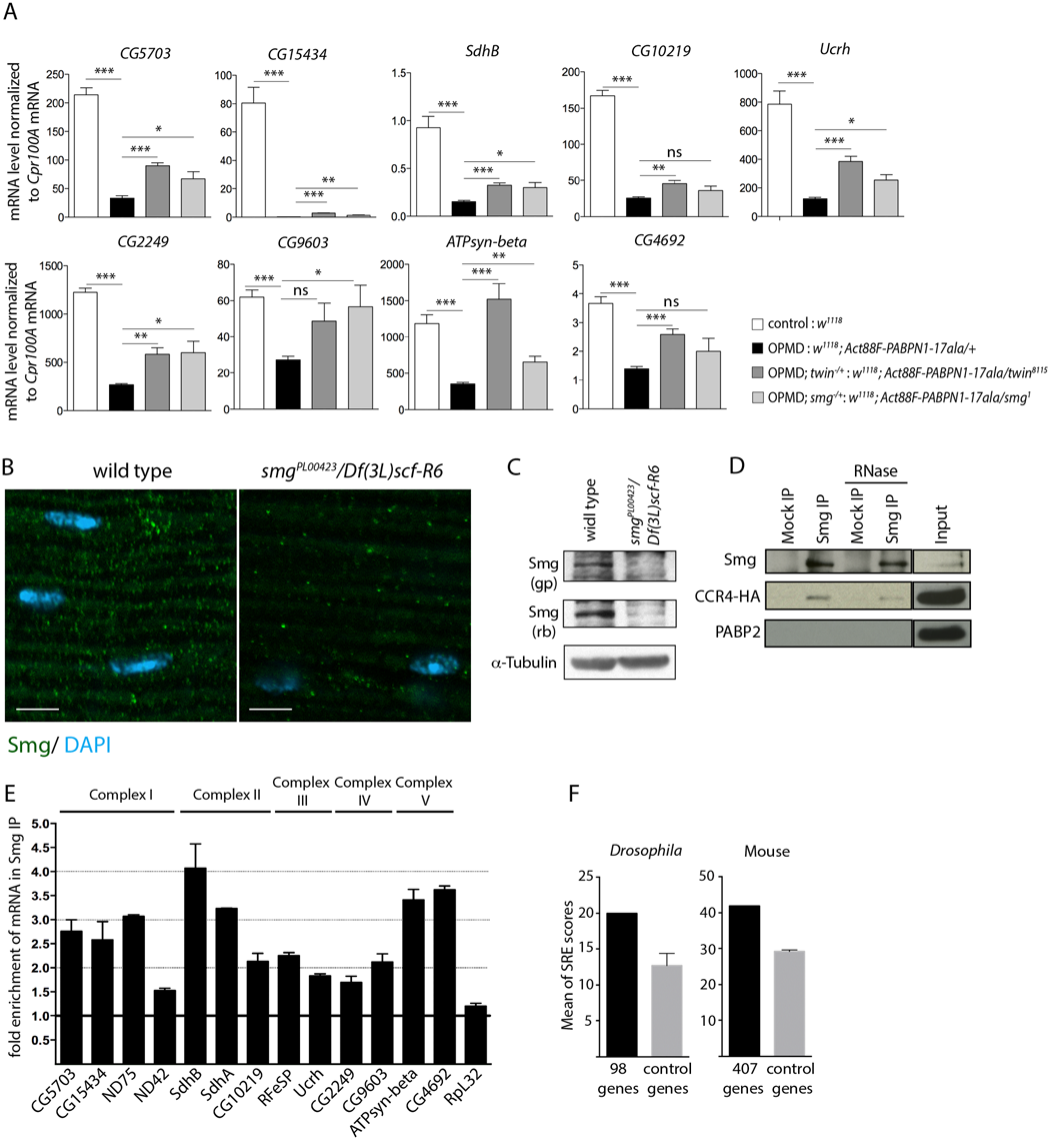
Smg binds to mRNAs encoding mitochondrial proteins and is involved in mRNA down-regulation due to PABPN1-17ala expression. A) Quantification of mRNA levels in control and PABPN1-17ala-expressing thoraxes in the presence or absence of heterozygous *twin* or *smg* mutations at day 6, using RT-qPCR. mRNA levels were normalized to *Cpr100A* mRNA. Means of two biological replicates quantified three times. Error bars represent standard deviation. * *p*-value <0. 05, ** *p*-value <0.01, *** *p*-value <0.001, ns: not significant, using the Student’s t-Test. B) Confocal images of immunostaining of indirect flight muscles from wild type and *smg* mutant (*smg* ^*PL00423*^ */Df(3L)scf-R6*) adult flies with anti-Smg (green). DNA was visualized with DAPI (blue). Smg protein levels were strongly reduced in *smg* ^*PL00423*^ */Df(3L)scf-R6* muscles compared to wild-type muscles. Scale bars: 5 μm. C) Western blots of protein extracts from wild-type and *smg* mutant thoraxes revealed with guinea pig (gp) and rabbit (rb) anti-Smg, showing the presence of Smg and its lower level in *smg* mutant. α-Tubulin was used as a loading control. D) Smg immunoprecipitations (IP) in *UASp-CCR4-HA/Mhc-Gal4* adult thoraxes, either in the presence or the absence of RNase A. Mock IP was with rabbit IgG. Input is the protein extract prior to immunoprecipitation. Western blots revealed with anti-Smg, anti-HA and anti-PABP2, showing CCR4-HA co-precipitation and the lack of PABP2 co-precipitation. E) Quantification of mRNA enrichment in Smg IP using RT-qPCR. The ratio of mRNA/control mRNA was set to 1 in the mock IP (black line). Normalization was with *sop* mRNA. *RpL32* mRNA is a negative control, which is not enriched in Smg IP. Means are from two independent IP quantified three times. Error bars represent standard error to the mean. F) Means of SRE scores of mRNAs down-regulated in PABPN1-17ala-expressing muscles and annotated with the term "mitochondrion" compared to those of control mRNAs, in *Drosophila* (left panel) and mouse (right panel). Twenty times 98 *Drosophila* and 10 times 407 mouse control genes were used. Error bars represent standard deviation.

The cleavage defect in PABPN1-17ala-expressing muscles appears to be general, yet the poly(A) tail shortening and destabilization occur on specific mRNAs. This indicates that the specificity of mRNA destabilization would not depend on the first defect in pre-mRNA cleavage, but on a downstream process. This process might correspond to deadenylation which can be activated on specific mRNAs through the recruitment of the CCR4-NOT deadenylation complex to these mRNAs by specific RNA binding proteins. In this model, OPMD would result from an altered function of PABPN1 leading to a reduced efficiency of nuclear cleavage/polyadenylation, then followed by normal active deadenylation of specific mRNAs. The reduced efficiency in nuclear cleavage would only affect the steady-state levels of mRNAs that are actively deadenylated. To test this model, we analysed the potential rescue of the levels of mRNAs that were decreased in *Drosophila* muscles expressing PABPN1-17ala, by *twin* heterozygous mutation (*twin* encodes the CCR4 deadenylase). Among the 20 mRNAs that we analysed using RT-qPCR, 18 showed higher expression levels in muscles expressing PABPN1-17ala in the presence of the *twin* mutant (Fig. 5A, S4A, B Fig).

The CCR4-NOT deadenylation complex itself has no specificity for particular mRNAs. The specificity instead depends on RNA binding proteins which interact with pools of mRNAs and recruit the deadenylation complex through direct protein interactions. Smg and Pum that were identified as suppressors of wing posture defects (Fig. 3B) are such RNA binding proteins. We tested the rescue of mRNA levels by RT-qPCR in muscles expressing PABPN1-17ala, in the presence of *pum* or *smg* heterozygous mutations. No rescue was observed with the *pum* mutant (*Act88F-PABPN1-17ala/pum* ^*3*^) (n = 19 mRNAs), suggesting that the phenotypic rescue of wing posture with this mutant did not involve the stabilization of these mRNAs, and might potentially involve their localization or translational regulation. Indeed, Puf3p, a yeast Pum homologue specifically binds to mRNAs encoding mitochondrial proteins and regulate their deadenylation or localization to mitochondria, coupled to translation [46–48].

In contrast, the levels of 14 mRNAs out of 20 analysed were increased in the presence of the heterozygous *smg* ^*1*^ mutation (Fig. 5A, S4A, B Fig). This suggests that Smg plays a role in the specific deadenylation and destabilization of mRNAs encoding mitochondrial proteins. We checked that the phenotypic rescue with *twin* and *smg* mutants did not involve the reduction of PABPN1-17ala levels (S4C, D Fig).

We analysed Smg expression in adult muscles. Smg is expressed in early embryos where it is cytoplasmic and accumulates in discrete foci that have been linked to deadenylation and/or translational repression [25,49], and it was recently shown to be present in larval muscles [50]. Using immunostaining and western blots, we validated the presence of Smg in cytoplasmic foci in adult thoracic muscles (Fig. 5B, C). The specificity of the antibody was verified using a *smg* mutant background. In addition, Smg was able to co-precipitate the CCR4 deadenylase in adult muscles (Fig. 5D). The co-precipitation was maintained in the presence of RNase A, indicating that Smg and CCR4 could form a complex independently of RNA. In contrast, PABP2 which accumulates in the nucleus was not co-precipitated with Smg (Fig. 5D).

We then performed Smg immunoprecipitation experiments in muscles and quantified mRNA enrichment using RT-qPCR, to address whether Smg could be in complex with mRNAs down-regulated in muscles expressing PABPN1-17ala. A number of these mRNAs encoding mitochondrial proteins were found to be enriched in Smg immunoprecipitations (Fig. 5E, S4E Fig). Smg binds to stem-loop structures, the SREs in which the consensus motif in the loop is CNGGN_0-4_ [51]. We analyzed potential SRE enrichment in genes down-regulated in muscles expressing PABPN1-17ala that were annotated with the term "mitochondrion" (98 genes, S2 Table), by calculating SRE scores in their mRNAs [51]. SRE sequences were enriched in these mRNAs compared to in control mRNAs (Fig. 5F).

These results suggest a direct association of mRNAs encoding mitochondrial proteins with Smg, leading to their CCR4-mediated deadenylation and destabilization.

### Rescue of PABPN1-17ala-induced phenotypes by reducing deadenylation does not involve decreased PABPN1 aggregation

**Fig 6:**
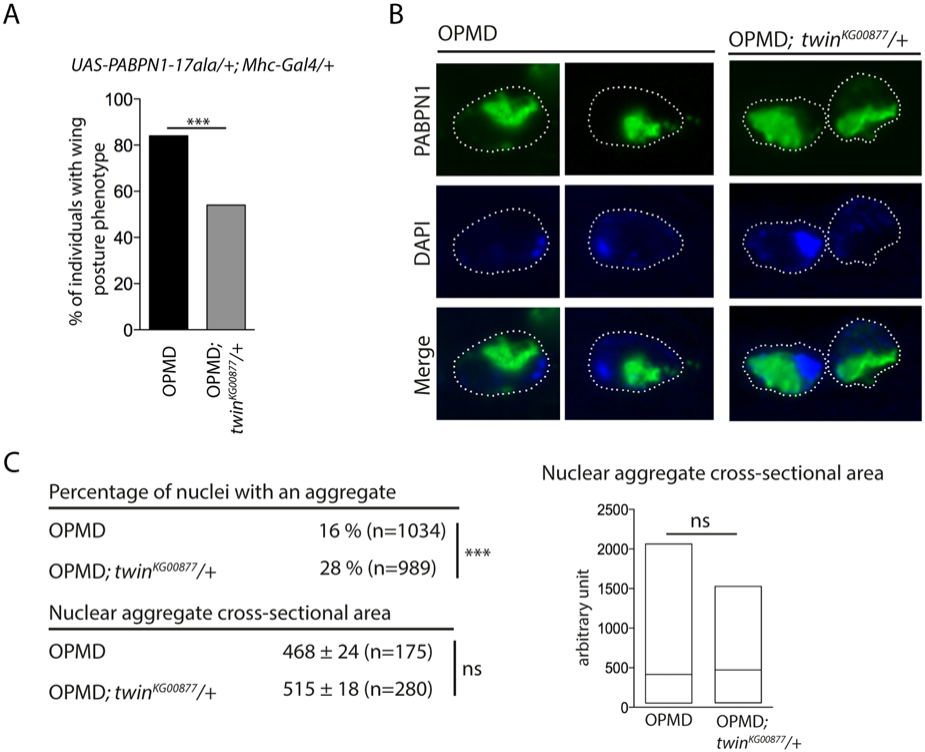
Nuclear PABPN1 aggregates in the OPMD *Drosophila* model. A) Genetic rescue of wing position phenotypes with *twin* ^*KG00877*^ hererozygous mutant. Percentage of wing posture defects in the presence or absence of *twin* ^*KG00877*^/+, scored at day 6. OPMD flies were *w* ^*1118*^ *; UAS-PABPN1-17ala/+; Mhc-Gal4/+* raised at 18°C. *** *p*-value <0.0001, using the χ^2^ test. B) Immunostaining of indirect flight muscles from OPMD (*w* ^*1118*^ *; UAS-PABPN1-17ala/+; Mhc-Gal4/+)* and OPMD; *twin* ^*KG00877*^ */+* (*w* ^*1118*^ *; UAS-PABPN1-17ala/+; Mhc-Gal4/twin* ^*KG00877*^) adult flies at day 6 with anti-PABPN1 (green), showing nuclear aggregates. DNA was visualized with DAPI (blue). Nuclei are outlined with a dotted line. C) Quantification of PABPN1 nuclear aggregates. (Top) Percentages of nuclei with a nuclear PABPN1 aggregate in OPMD and OPMD; *twin* ^*KG00877*^ */+* indirect flight muscles (genotypes as in B) *** *p*-value <0.0001, using the χ^2^ test. (Bottom) Quantification of nuclear aggregate areas. Each nuclear aggregate was delimited in a focal plan and the surface was calculated using ImageJ. Mean values of the areas with standard errors of the mean are indicated in arbitrary units Distribution of cross-sectional areas is shown as box plots (right), the median is indicated as an horizontal line within the box. ns: not significant, using the Student’s t-Test. Thoracic muscles were stained with anti-PABPN1 and DAPI and nuclear aggregates were visualized and scored using both staining. Quantification was from three independent experiments.

The formation of nuclear PABPN1 aggregates is a hallmark of OPMD, although the presence of nuclear aggregates and muscle defects can be uncoupled in animal models [23,52]. Hitherto, components that were identified as decreasing muscle degeneration in the *Drosophila* model also reduced PABPN1 aggregation, since these components either directly interacted with PABPN1 (anti-PABPN1 intrabody), or with protein aggregation [22,26]. We asked whether the rescue of PABPN1-17ala-induced phenotypes by affecting poly(A) tail length regulation might interfere with PABPN1 aggregate formation. Nuclear aggregates have been monitored previously in *UAS-PABPN1-17ala/+; Mhc-Gal4/+ Drosophila* muscles [22,23], we therefore checked that a *twin* heterozygous mutant decreased the wing posture defects in such flies (Fig. 6A). PABPN1 nuclear aggregates were then quantified and their surface was measured in thoracic muscles of flies expressing PABPN1-17ala, in the presence or absence of *twin* heterozygous mutation. Strikingly, although the surface of the aggregates was not significantly different in both conditions, the number of nuclei containing an aggregate increased in the presence of *twin* heterozygous mutant (Fig. 6B, C). Therefore, in contrast to previously described conditions of rescued muscle function in PABPN1-17ala-expressing muscles, here improvement of muscle function did not correlate with reduced PABPN1 aggregation.

These results are consistent with other data reporting the dissociation between the presence of PABPN1 nuclear aggregates and muscle defects, and suggest that PABPN1 aggregates are not always causative of muscle defects.

### Early down-regulation of mRNAs encoding mitochondrial proteins is conserved in the OPMD mouse model

**Fig 7:**
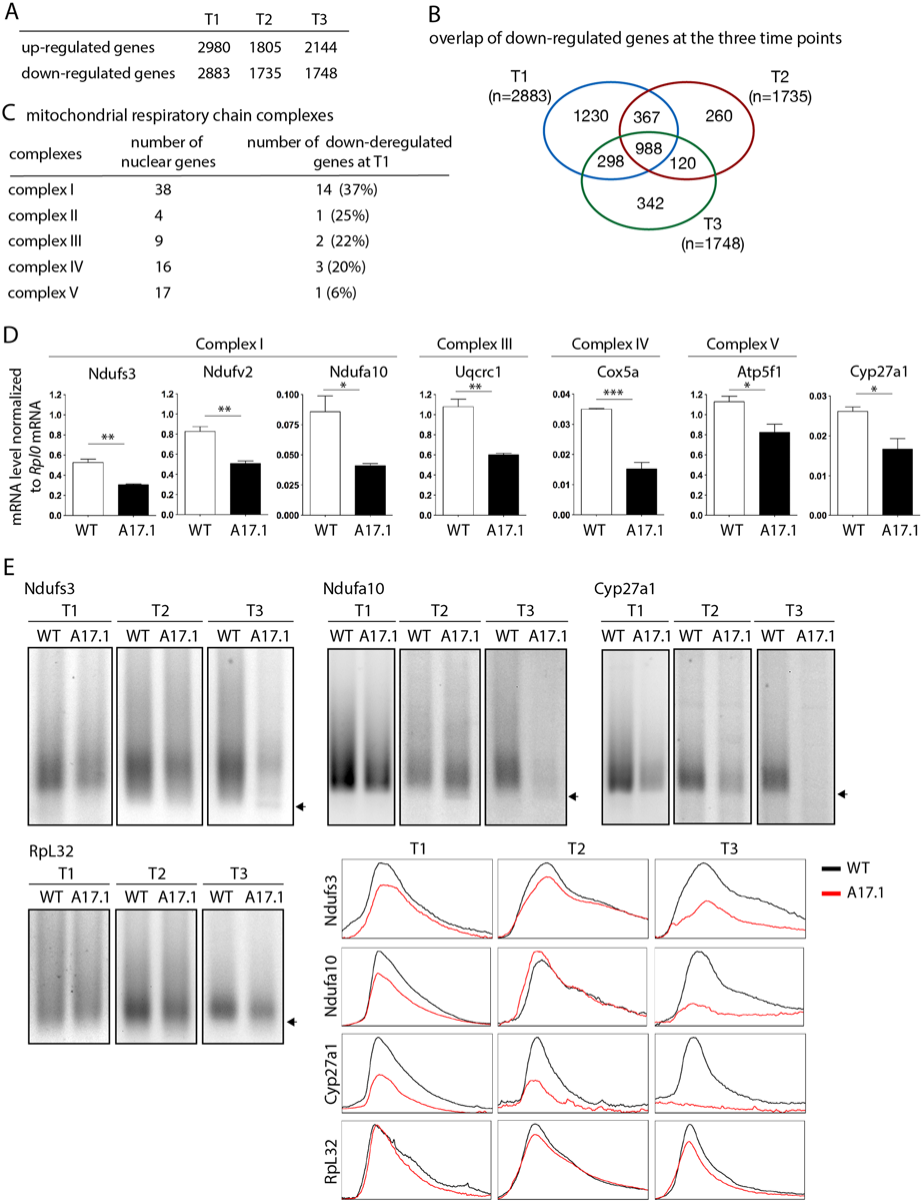
Deregulation of the mitochondrial pathway in the OPMD mouse model. A) Number of deregulated genes in A17.1 mouse skeletal muscles using microarrays. T1, 6 weeks; T2, 18 weeks; T3, 26 weeks. B) Venn diagram of overlapping down-regulated genes at T1, T2 and T3. C) Number of nuclear genes encoding mitochondrial respiratory chain complex subunits down-regulated in A17.1 mouse muscles. D) Quantification of levels of mRNAs encoding mitochondrial respiratory chain subunits in control (WT) and A17.1 quadriceps skeletal muscle at T1, using RT-qPCR. Normalization was with *Rplp0* mRNA. Means are from three biological replicates, error bars represent standard deviation. * *p*-value <0.05, ** *p*-value <0.01, *** *p*-value <0.001, using the Student’s t-Test. E) ePAT assays of mRNAs encoding mitochondrial proteins in control (WT) and A17.1 quadriceps skeletal muscles. Arrows indicate poly(A) tails of 12A. Accumulation of 12A poly(A) tails was visible in A17.1 muscles at T2 and/or T3. *RpL32* is a negative control mRNA encoding a ribosomal protein. Profiles of ePAT assays using the ImageJ software are shown.

To extend our study to a mammalian model, quadriceps gene expression using microarrays was compared between control mouse (FvB) and A17.1 mouse which expresses PABPN1-17ala in skeletal muscle [53], at three time points (T1, 6 weeks; T2, 18 weeks; and T3, 26 weeks) [54]. Up- and down-regulated genes were found at all time points (Fig. 7A), with the ubiquitin-proteasome system being higly deregulated [9]. Annotation clustering enrichment analysis using DAVID [55] revealed that down-regulated genes common to all three time points were mostly enriched in genes encoding mitochondrial proteins (GO:0005739 "mitochondrion", Fold enrichment 14.8, *p*-value 5.88E-23), and we identified several nuclear genes involved in oxidative phosphorylation that were down-regulated, starting at the earliest time point (Fig. 7B, C, S3 Table). RT-qPCR confirmed the down-regulation observed with microarrays at T1 (Fig. 7D). These data obtained on pre-symptomatic muscles, for which no muscle weakness was evidenced [53], further confirmed the down-regulation of mRNAs encoding mitochondrial proteins as an early defect in OPMD. Using ePAT (extension PAT) assays, we found that the down-regulation of these mRNAs correlated with the accumulation of shorter poly(A) tails (Fig. 7E).

SREs are conserved from yeast to mammals [56,57]. We searched for the presence of potential SREs in the genes down-regulated at T1 that were annotated with the term "mitochondrion" (407 genes, S4 Table) [51]. SRE scores were higher in these down-regulated mRNAs than in control mRNAs (Fig. 5F) indicating an enrichment of SREs in mRNAs down-regulated in muscles from the A17.1 mouse.

In order to validate the role of SREs in the regulation of mRNAs that were down-regulated in PABPN1-17ala expressing muscles, we selected Ndufa10 mRNA which contains a potential SRE in its 3'UTR (S5A Fig). We took advantage of the HEK293T human cell line which we found expressing SAMD4A, the human homologue of Smg, using RT-PCR, western blot and immunostaining (S5B, C Fig). Reporter constructs containing Ndufa10 3'UTR either with the potential SRE or with a mutant form of this element were used for transfection, and mRNA levels produced from these constructs were quantified using RT-qPCR. Mutation of the SRE resulted in higher levels of mRNA (S5A Fig), consistent with the role of the SRE in mRNA destabilization.

We conclude that the down-regulation of mRNAs encoding mitochondrial proteins in pre-symptomatic muscles expressing PABPN1-17ala is conserved in mouse. This down-regulation correlates with accumulation of shorter poly(A) tail lengths of these mRNAs and with their enrichment in SREs.

### Mitochondrial protein levels are decreased in OPMD patient muscles

**Fig 8:**
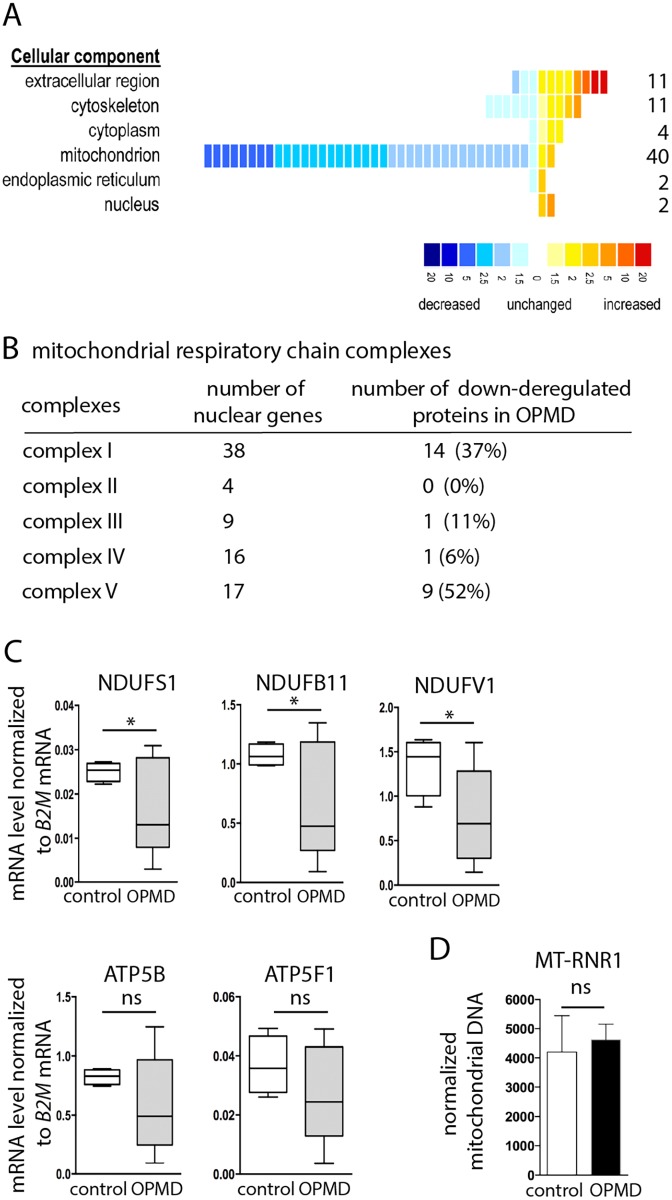
Decrease of mitochondrial protein levels in human OPMD muscle biopsies. A) Distribution of differentially expressed proteins from OPMD and control human muscle samples, following a quantitative label-free LC-MS profiling proteomic analysis. Proteins are sorted according to their GO terms in "cellular component" using DAVID. Each square represents a protein. The value of the fold change is color-coded. Yellow to red, over-expressed in OPMD samples (maximum 20-fold); blue, under-expressed in OPMD samples (minimum -20-fold). B) Number of subunits of the mitochondrial respiratory chain complexes down-regulated in OPMD patient muscle biopsies. C) Quantification of levels of mRNAs encoding mitochondrial respiratory chain subunits in control and OPMD patient muscle biopsies, using RT-qPCR. Normalization was with B2M (Beta-2 microglobulin) mRNA. Box plots are from 4–7 muscle biopsies, the median is indicated as an horizontal line within the box. * *p*-value <0.05 using the Student’s t-Test. For ATP5B and ATP5F1, the median is lower in OPMD than in control biopsies, although the difference is not recorded as significant. D) Quantification of mitochondrial DNA on muscle biopsies, using qPCR. The MT-RNR1 mitochondrial gene was quantified. Normalization was with B2M nuclear DNA. Means are from four biological replicates, error bars represent standard deviation. ns: not significant, using the Student’s t-Test.

To further validate a defect in mitochondrial components in OPMD patients, we used a proteomic approach on control and OPMD patient sternocleidomastoid muscle biopsies. This muscle, which is clinically unaffected [58] was used as a surrogate for pre-symptomatic samples. Strikingly, among the 70 proteins that were found significantly deregulated, 49 were down-regulated and 78% of them were mitochondrial proteins (n = 38) (Fig. 8A, B, S5 Table). Moreover, 53% (n = 20) and 39% (n = 15) of these proteins were orthologous to mitochondrial proteins encoded by down-regulated mRNAs in the *Drosophila* and mouse models, respectively. We verified by RT-qPCR that the corresponding mRNAs were down-regulated in OPMD patient biopsies (Fig. 8C), whereas the amount of mitochondrial DNA remained unchanged (Fig. 8D).

These results confirm the molecular data obtained in both animal models, showing the down-regulation of mRNAs encoding mitochondrial proteins as an early defect in OPMD. They further show the down-regulation of mitochondrial proteins and are consistent with mitochondrial dysfunction playing an important role in early stages of OPMD progression.

## Discussion

**Fig 9:**
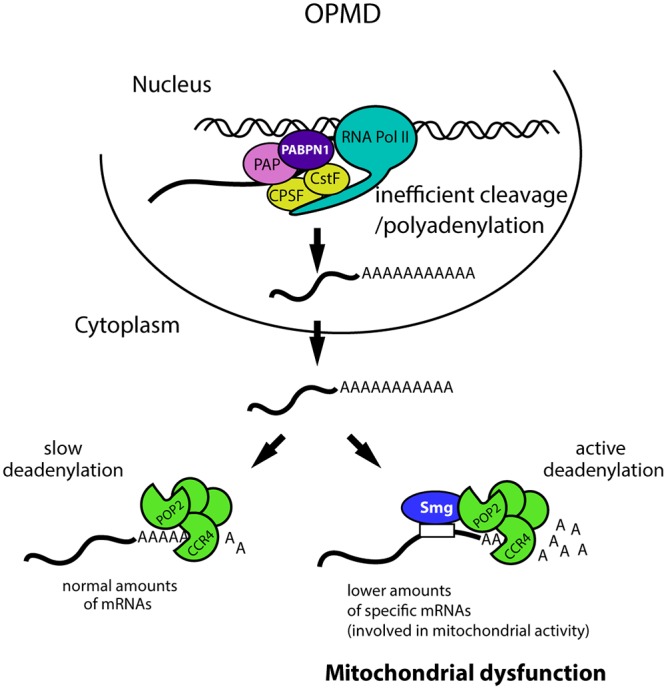
Molecular model of OPMD. The first molecular defect in OPMD would be a general decrease in the cleavage/polyadenylation reaction resulting from affected PABPN1 function. This would not lead to a reduction of mRNA levels at steady-state for most mRNAs, but would lead to such a decrease for mRNAs actively deadenylated by Smg/CCR4-NOT, among which mRNAs involved in mitochondrial function. This would result in mitochondrial dysfunction and in turn affected muscle function. Additional mechanisms of mRNA regulation occurring downstream of the first defect in cleavage/polyadenylation are also expected to be involved. CPSF, Cleavage and polyadenylation specificity factor; CstF, Cleavage stimulation factor; PAP, poly(A) polymerase.

The molecular defects underlying OPMD pathology remain largely undetermined, although recent advances have implicated apoptosis [11] and a general deregulation of the ubiquitin-proteasome system [9]. However, these are downstream events in the pathogenesis. Here, we further investigate the molecular mechanisms involved by analysing early defects in the disease. We show that specific mRNAs encoding proteins involved in mitochondrial activity are present at lower levels in pre-symptomatic OPMD muscles; this reduced expression results from the shortening of their poly(A) tails which leads to their destabilization. Poly(A) tail length regulation plays a key role in OPMD since muscle function is improved when deadenylation is decreased using mutants. We further show that nuclear cleavage/polyadenylation of pre-mRNAs is inefficient in PABPN1-17ala-expressing muscles (Fig. 9). This defect occurs both on genes which are and on those which are not down-regulated, indicating that it does not *per se* systematically lead to reduced steady-state mRNA levels. The decreased levels of specific mRNAs result from their active deadenylation, itself dependent, at least in part, on the specific interaction of these mRNAs with the Smg RNA binding protein and the recruitment by Smg of the CCR4-NOT deadenylation complex (Fig. 9). In addition, our genetic data revealing the potential involvement of other RNA binding proteins suggest that OPMD pathogenesis is complex and probably involves additional mechanisms of RNA regulation.

The function of PABPN1 during cleavage/polyadenylation has been documented. Nuclear polyadenylation occurs in two steps: first, cleavage of the pre-mRNA at the poly(A) site, which is co-transcriptional, and second, polyadenylation which potentially occurs after dissociation of the RNA from the RNA polymerase II. PABPN1 was shown to be involved in the second step, polyadenylation, for the control of poly(A) tail lengths [14,15]. More recent data have also implicated PABPN1 in the cleavage step for the regulation of weak poly(A) sites [20]. Here we show impaired cleavage at poly(A) sites in the *Pabp2* loss-of-function mutant, revealing a more general role of PABP2/PABPN1 in this step of the reaction. In the regulation of weak poly(A) sites, PABPN1 binds to non-canonical polyadenylation signals and prevents the binding of CPSF (Cleavage and polyadenylation specificity factor) required for cleavage [20]. A general function of PABPN1 in cleavage would require other interactions, for example with proteins required for cleavage, such as the poly(A) polymerase known to associate with PABPN1 [14]. Although a global shift to proximal poly(A) sites has been reported in the mouse model of OPMD [20,21], the down-regulation and poly(A) tail shortening of specific mRNAs that we functionally show to participate in OPMD pathogenesis are independent of alternative poly(A) site utilization.

The molecular defects observed in PABPN1-17ala-expressing muscles, reduction of mRNA poly(A) tail length and decreased efficiency of cleavage at poly(A) sites are similar to those observed in *Pabp2* loss-of-function mutants ([16], this study). This suggests that part of the defects in OPMD could result from partial *PABPN1* loss-of-function. However, the genetic suppression of wing posture phenotypes by reducing the dosage of *Pabp2* does not favour a simple loss-of-function model. It has been proposed for polyglutamine diseases that the pathogenesis could result from both the gain-of-function and the loss-of-function of the same protein [59]. The protein responsible for the disease would exist in two different conformations with two different yet normal functions. Extension of the polyglutamine tract would favour one conformer resulting in increased amounts of this conformer and the partial loss-of-function of the other conformer; the pathology would result from both these effects. In this model, the mutant protein would have the same function as the normal protein but would have the ability to alter the balance between both protein forms. Several properties of PABPN1 are consistent with this model for OPMD. We have shown previously that the normal function of PABPN1 and more specifically its RNA binding activity is required for OPMD-like defects in the *Drosophila* model [23]. In addition, PABPN1-17ala half-live was reported to be longer than that of PABPN1 in cell models, leading to higher accumulation of PABPN1-17ala and protein aggregation [60]. Thus, expansion of the polyalanine tract results in protein "overexpression" which contributes to the pathology. Given this data, overepression of the normal protein might be expected to induce similar defects as expression of the mutant protein, as it is the case in *Drosophila* models for other disorders [61]. Consistent with this, we previously reported that PABPN1 expression in *Drosophila* muscles induced wing posture defects, although at lower levels than PABPN1-17ala expression [23]. Finally, normal PABPN1 is also known to form oligomers during nuclear polyadenylation and can form nuclear aggregates that recruit ubiquitin and proteasomes under specific physiological conditions [52,62].

Because the presence of nuclear aggregates and muscle defects can to some extent be uncoupled, we have previously proposed that nuclear aggregates are not always pathological [23]. This is consistent with results concerning polyglutamine diseases where aggregates can have a protective role [63]. We find that the improvement of muscle function when deadenylation is genetically reduced correlates with an increased number of PABPN1 aggregates, again strengthening the notion that the aggregates are not always causative of muscle defects. In that case, muscle protection that results from the reduction of molecular defects could allow the formation of more PABPN1 aggregates. Thus, these aggregates might not always be pathological, in particular during early stages of the disease, although they might become so at later stages, when their increased size could interfere with nuclear function.

A major conclusion from our study is that the specificity of the defect in OPMD does not depend *per se* on PABPN1 defect in pre-mRNA cleavage, but on Smg-dependent regulation occurring in the cytoplasm. Because of the shift to proximal poly(A) sites that correlated with mRNA up-regulation, described in the OPMD mouse model [20], we asked whether a similar mechanism could lead to increased Smg levels in *Drosophila* muscles expressing PABPN1-17ala and underlie increased deadenylation. However, the same poly(A) site was used in normal and PABPN1-17ala-expressing muscles, and we failed to detect major deregulation of *smg* mRNA and protein levels in PABPN1-17ala-expressing muscles (S6A-C Fig). We propose that normal Smg-dependent deadenylation, following inefficient pre-mRNA cleavage, could lead to the reduced levels of specific mRNAs that we observe (Fig. 9). In addition, other processes such as mRNP remodelling could contribute to enhanced mRNA decay in the course of the disease progression. Indeed, Smg forms cytoplasmic foci which are distinct, but related to other cytoplasmic RNA granules such as processing (P) bodies or stress granules, in which mRNAs are degraded or translationally repressed, and the regulation of which affects mRNA regulation [25,64].

A recent study also revealed the implication of Smg/SAMD4A in Myotonic Dystrophy Type 1 (DM1). In that case, Smg mechanism of action appeared to be different, since overexpression of Smg decreased DM1 muscle defects by reducing unproductive CUGBP1-eIF2α translational complexes [50].

Mitochondrial dysfunction has been shown to play a major role in most neurodegenerative diseases including Parkinson's, Alzheimer's, Huntington's and other polyglutamine diseases [65]. More recent data have uncovered that aside mitochondrial function in energy production, mitochondrial dynamics including trafficking and quality control is instrumental in pathogenesis [65–67]. Mitochondria also have a key role in muscle function. *Drosophila* mutants of *pink1* and *parkin*, mutations of which cause Parkinson's disease in man, lead to mitochondrial dysfunction and flight muscle degeneration [68,69]. We show that mitochondrial dysfunction is also an important component of OPMD: Muscle function is improved when mitochondrial biogenesis and activity are genetically increased; in addition, mitochondrial proteins are down-regulated in OPMD muscle biopsies from patients. We identify the molecular defects leading to early mitochondrial dysfunction in OPMD: mRNAs encoding mitochondrial proteins are down-regulated due to their Smg-dependent deadenylation. Therefore, our data reveal Smg as a regulator of mRNAs involved in mitochondrial function. This finding might have important implications on the role of Smg in several neurodegenerative diseases that involve mitochondrial dysfunction and/or RNA toxicity.

## Materials and Methods

### Ethics statement

Experimental animal studies were conducted under approval of both the RHUL Animal Research Ethics Committee, and the UK Home Office, and with a UK Home Office license (PPL 70/7008) under the UK Animals (Scientific Procedures) Act 1986. Animal were euthanized by an approved schedule 1 procedure under these statutory regulations. All human muscle biopsies were obtained during surgical procedure after informed consent in accordance with the French legislation on ethical rules.

### RNA, DNA, quantitative PCR and Poly(A) test assays

Total RNA was prepared from 10 *Drosophila* thoraxes, mouse quadriceps, human muscle frozen biopsies or HEK293T cells, using Trizol (Invitrogen) as recommended by the manufacturer. DNA was digested using TURBO DNase (Ambion) or DNase RNase free (Qiagen). Total RNA concentration was determined with nanodrop ND-1000 spectrophotometer. For RT-qPCR, 0.1–1μg of total RNA was reverse transcribed with SuperScript III (Invitrogen). Random hexamers (Roche) were used for reverse transcription following Smg immunoprecipitations and for pre-mRNA cleavage anlysis. Oligo-d(T)_12-18_ primers (Invitrogen) were used in RT-qPCR comparing mRNA levels in *Drosophila* thoraxes of different genotypes; we verified that utilization of random hexamers for these RT-qPCR reproduced the deregulation observed with oligo-d(T)_12-18_ primers (S1B Fig). A mix of oligo-d(T)_12-18_ primers and random hexamers was used for reverse transcription performed on mouse and human RNAs. RNA levels were calculated using the LightCycler 480 SYBR Green I Master (Roche) on the LightCycler 480 Instrument (Roche), and normalized with *Drosophila sop* and/or *Cpr100A* mRNAs, mouse *Rplp0* and human B2M. *sop* and *Rplp0* encode ribosomal proteins, β2-microglobulin (B2M) encodes a component of MHC class I molecules, and *Cpr100A* encodes a cuticular protein present in thorax cuticle but not expressed in muscles. Poly(A) test (PAT) assays were performed with 1μg of total RNA using either regular PAT [70] (Fig. 3C), or ePAT [71] (Fig. 7E, S3, S6 Fig) methods. Briefly, for the PAT reaction, mRNA poly(A) tails were coated with oligo-d(T)_12-18_ primers which were then ligated; this reaction was followed by annealing of the d(T)-anchor primer to the overhanging remaining As at 12°C and its subsequent ligation, then by reverse transcription from this ligated primer, and PCR using d(T)-anchor and a gene specific primer [70]. For ePAT, the d(T)-anchor primer was annealed to mRNA poly(A) tails at 25°C and used as template for mRNA extension with Klenow polymerase (see S6A Fig, ePAT); this reaction was then switched to 55°C to dissociate annealings that had not been extended by Klenow polymerase, and followed by reverse transcription and PCR using d(T)-anchor and a gene specific primer [71]. PCR fragments were visualized on 2% agarose gel. RNase H digestion was performed on 3μg RNA with 5 Units RNase H (Biolabs) in the presence or not of 1μg oligo-d(T)_12-18_. Mitochondrial DNA (mtDNA) was extracted using a standard DNA extraction method. mtDNA was isolated from 5 thoraxes per genotype for *Drosophila*, and from 100 5μm-thick cryosections for human muscle biopsies. Total DNA concentration was quantified with the nanodrop ND-1000 spectrophotometer and 0.4 ng of total DNA was used for qPCR. *Drosophila* mtDNA content was quantified using *mt*:*CoI*, *mt*:*CoII*, and *mt*:*cyt-b* genes, and normalized with *RpL32* genomic DNA. mtDNA content in human muscle biopsies was quantified using the MT-RNR1 gene and normalized using B2M nuclear genomic DNA. Primers used are indicated in S1 Text.

### Enzymatic activities of mitochondrial complexes

Enzymatic activities were measured from homogenates of 10 thoraxes prepared at 4°C in 700 μl of phosphate buffer (50 mM; pH 7). Enzymatic activities for each complex were measured from five independent homogenates per genotype. Activities of complexes I, II, II+III and IV, as well as citrate synthase activity were determined spectrophotometrically from the supernatant fraction as described previously [72]. Protein concentrations were measured using the Bio-Rad protein assay kit (Bio-Rad).

### Antibodies, western blots, immunostaining and immunoprecipitations

Anti-Smg antibody was raised in rabbit against amino acids 571 to 771 of the Smg protein (SAM domain). Briefly, this fragment was expressed in *E*. *coli* as a His6-SUMO fusion protein. After metal affinity chromatography, the N-terminal SUMO domain was cleaved with ULP protease, the two protein fragments were separated by a second metal affinity column, and the SAM domain of Smg was finally purified by Superdex75 gel filtration. The antibody was produced at Eurogentec, using four injections of 100 μg of Smg SAM domain. Protein extracts were obtained from 5 or 10 thoraxes per genotype. Western blots were performed as described [73]. Immunostaining of thoracic muscles were performed as described previously [23]. Antibodies used for western blots, immunostaining and Smg immunoprecipitation procedures are indicated in Supporting Materials and Methods.

### Calculation of SRE scores

SRE scores were determined as reported [51] using 98 *Drosophila* and 407 mouse genes down-regulated in OPMD muscles and annotated with the term "mitochondrion". Control gene lists were generated by random selection within *Drosophila* genes found not to be enriched in Smg immunoprecipitations, with a fold change ≤1 [51] (98 genes, 20 times), and all mouse genes (*Mus musculus*, Ensembl) (407 genes, 10 times), respectively. Genes present in the tested lists were removed from the control lists. SRE scores were determined for all potential transcripts of each gene and the highest score only was considered per gene.

## Supporting Information

S1 Text Supporting materials and methods. (PDF)

S1 Fig Quantification of mRNAs encoding mitochondrial respiratory chain subunits and of negative control mRNAs. A) Quantification of mRNA levels in control and PABPN1-17ala-expressing adult thoraxes at day 2, using RT-qPCR. mRNA levels were normalized to *sop* mRNA. Means are from three biological replicates, error bars represent standard deviation. * *p*-value <0.05, ** *p*-value <0.01, *** *p*-value <0.001, ns: not significant, using the Student’s t-Test. Genotypes are indicated for (A-C). B) Quantification of mRNA levels at day 2, using RT-qPCR, with reverse transcription performed with random hexamers, showing down-regulation in PABPN1-17ala-expressing thoraxes compared to control. mRNA levels were normalized to *sop* mRNA. Means are from two biological replicates quantified three times, error bars represent standard deviation. ** *p*-value <0.01, *** *p*-value <0.001, using the Student’s t-Test. C) Quantification of negative control mRNAs which were found unaffected in microarray analysis, using RT-qPCR. mRNA levels were normalized to *sop* mRNA. Means are from two biological replicates quantified three times, error bars represent standard deviation. ns: not significant, using the Student’s t-Test.
(PDF)

S2 Fig *GstD1-GFP* expression in OPMD muscles. A) GFP expression in indirect flight muscles of control (*GstD1-GFP/+; Mhc-Gal4/+*) and OPMD (*UAS-PABPN1-17ala*, *GstD1-GFP/+; Mhc-Gal4/+*) flies at day 2. Expression of the *GstD1-GFP* transgene was visualized by direct fluorescence and captured with the same settings for both genotypes. B) Western blots of control (*GstD1-GFP/+; Mhc-Gal4/+*) and OPMD (*UAS-PABPN1-17ala*, *GstD1-GFP/+; Mhc-Gal4/+*) adult thoraxes at day 6 to quantify GstD1-GFP protein. Protein extracts were from 0.25 thoraxes of flies raised at 18°C. α-Tubulin was used as loading control.
(PDF)

S3 Fig Affected mRNA poly(A) tails in the OPMD *Drosophila* model. A) ePAT (extension PAT) assays of mRNAs encoding mitochondrial proteins in control (*w* ^*1118*^) and OPMD (*Act88F-PABPN1-17ala/+*) adult thoraxes at day 6. *sop* mRNA was used as a negative control. Arrows indicate poly(A) tails of 12A. B) Profiles of ePAT assays shown in A, using ImageJ software.
(PDF)

S4 Fig Role of Smg in the OPMD *Drosophila* model. A) Quantification of mRNA levels in control and PABPN1-17ala-expressing thoraxes in the presence or absence of heterozygous *twin* or *smg* mutations at day 6, using RT-qPCR. mRNA levels were normalized to *Cpr100A* mRNA. Means of two biological replicates quantified three times. Error bars represent standard deviation. * *p*-value <0. 05, ** *p*-value <0.01, *** *p*-value <0.001, ns: not significant, using the Student’s t-Test. Genotypes are indicated for (A-D). B) Quantification of negative control mRNAs which were unaffected in PABPN1-17ala-expressing muscles in the microarray analysis, using RT-qPCR. mRNA levels were normalized to *sop* mRNA. *RpS6* mRNA levels remained unaffected in the different genotypes. The upregulation of *RpL32* mRNA in the *twin* ^*-/+*^ condition can be explained by its reduced deadenylation, since the CCR4-NOT complex can act without specificity. Means are from two biological replicates quantified three times, error bars represent standard deviation. *** *p*-value <0.001, ns: not significant, using the Student’s t-Test. C) Quantification of *PABPN1-17ala* mRNA in thoraxes at day 6 using RT-qPCR, either in the absence (*w* ^*1118*^ *; Act88F-PABPN1-17ala/+*) or the presence of *twin* (*w* ^*1118*^ *; Act88F-PABPN1-17ala/twin* ^*8115*^) or *smg* (*w* ^*1118*^ *; Act88F-PABPN1-17ala/smg* ^*1*^) mutations. mRNA levels were normalized to *sop* mRNA. Mean quantifications are from two biological replicates quantified three times. Error bars represent standard deviation. ns: not significant using the Student’s t-Test. D) PABPN1-17ala protein levels determined by western blots. Protein extracts were from 0.25 thoraxes at day 6. α-Tubulin was used as a loading control. E) Western blot validating Smg immunoprecipitation from thoraxes in the conditions used for RNA co-precipitations shown in Fig. 5E. The western blot was revealed with rabbit (rb) anti-Smg antibody.
(PDF)

S5 Fig SRE-dependent mRNA regulation in human cells. A) Quantification of mRNAs produced from SRE+ and SRE- reporter constructs transfected in HEK293T cells. (Top) Schematic representation of the 3'UTR of the Ndufa10 gene containing the SRE sequence (black circle, SRE+) or mutated in the SRE (open circle, SRE-), downstream of the Renilla Luciferase gene. Sequences of the SRE region are shown. Nucleotides in the SRE loop (CTGG) are in bold, those in the stem are underlined. The SRE mutation (SRE-) was designed to create a *Hpa*1 restriction site (red). (Bottom) HEK293T cells were co-transfected with either pRLTK-Luc-SRE+ or pRLTK-Luc-SRE-, and PGK-eGFP constructs. mRNA levels were quantified using RT-qPCR, normalized to B2M or GFP mRNA levels. The levels of GFP mRNA normalized to B2M mRNA were similar in both types of transfection (right panel). Means are from eight quantifications performed in three independent transfections, error bars represent standard deviation. * *p*-value <0.05, using the Student’s t-Test. B) RT-PCR (left) and western blot (right) showing that HEK293T cells express SAMD4A at the mRNA and protein levels. For RT-PCR, negative controls in which the reverse transcriptase was omitted (-RT) are shown. The western blot was revealed with anti-human SAMD4A. C) Confocal images of SAMD4A immunostaining in HEK293T cells with anti-human SAMD4A (red) showing accumulation of SAMD4A in foci. DNA was revealed with Hoechst (blue).
(PDF)

S6 Fig *smg* mRNA is not deregulated in PABPN1-17ala-expressing *Drosophila* muscles. A-B) Poly(A) site utilization in control and PABPN1-17ala-expressing muscles. A) Scheme of the experiment. RNAs were treated with RNase H either in the presence or the absence of oligo-d(T)_12-18_ to degrade the poly(A) tail (1), and then subjected to ePAT (2). Black arrows indicate the primers used in the ePAT reaction. The oligo(A) remaining of the poly(A) tail after RNase H digestion allows to produce a discrete band in PCR amplification which indicates the site used for polyadenylation. B) Results of the experiment described in (A). The same poly(A) site was used in both control and PABPN1-17ala-expressing muscles (arrow). Genotypes are indicated in (C). C) Quantification of *smg* mRNA and protein levels in control and PABPN1-17ala expressing muscles at day 6. Quantification of *smg* mRNA using RT-qPCR. mRNA levels were normalized to *sop* mRNA (left). Means are from two biological replicates quantified three times, error bars represent standard deviation. ns: not significant, using the Student’s t-Test. Western blot of control and PABPN1-17ala-expressing thoraxes revealed with rabbit anti-Smg (right). Protein extracts were from 5 thoraxes. α-Tubulin was used as a loading control.
(PDF)

S1 Table Down-regulated genes in *Drosophila* muscles expressing PABPN1-17ala. K-means clustering is from microarray analysis previously described [26].
(XLS)

S2 Table Down-regulated genes in *Drosophila* muscles expressing PABPN1-17ala, annotated with the GO term "mitochondrion". (XLSX)

S3 Table Functional annotation clustering of common down-regulated genes in A17.1 mouse muscles, from microarray analysis previously described [54]. (XLSX)

S4 Table Down-regulated genes in A17.1 mouse muscles at T1, annotated with the GO term "mitochondrion". (XLSX)

S5 Table Differentially expressed proteins in muscle biopsies from OPMD patients. (XLSX)
