# Supplementary material for: Mitochondrial Dysfunction Reveals the Role of mRNA Poly(A) Tail Regulation in Oculopharyngeal Muscular Dystrophy Pathogenesis
Source: PLoS Genet. 2015 Mar 27;11(3):e1005092. doi: 10.1371/journal.pgen.1005092 (PMC4376527; doi:10.1371/journal.pgen.1005092)
Supplement: S1 Text — (PDF) [file pgen.1005092.s001.pdf]

Mitochondrial dysfunction reveals the role of mRNA poly(A) tail regulation in oculopharyngeal muscular dystrophy pathogenesis

Aymeric Chartier[1]*, Pierre Klein[2]*, Stéphanie Pierson[1], Nicolas Barbezier[1], Teresa Gidaro[2], François Casas[3], Steven Carberry[4], Paul Dowling[4], Laurie Maynadier[1], Maëlle Bellec[1], Martine Oloko[2], Claude Jardel[5], Bodo Moritz[6], George Dickson[7], Vincent Mouly[2], Kay Ohlendieck[4], Gillian Butler-Browne[2], Capucine Trollet[2#] and Martine Simonelig[1#]

Supporting Information

Supporting Materials and Methods

Drosophila stocks

The w^{1118} strain was used as control. Transgenic stocks and mutants were *UAS-PABPN1-17ala* [23], *Mhc-Gal4* [74], *UASp-ccr4-HA* [24], *UAS-Delg-HA* [36], *GstD1-GFP* [29], $twin^{8115}$ [25], $twin^{KG00877}$ [40], smg^{1} and *Df(3L)scf-R6* [75], hrg^{PAP12} and hrg^{PAP21} [43], $wisp^{KG05287}$ [42], $Papb2^{55}$ [16], $pAbp^{k10109}$ [76], how^{18} [77], bic^{1} [78], $BicC^{AA4}$ [79], orb^{F343} [80,81], pum^{3} and pum^{9} [82], pum^{MSC} [83], pum^{01688} [84] and $Pop2^{DG14804}$, $spargel^{EY05931}$, $ewg^{EY05137}$, $dERR^{G4389}$, Rga^{03834}, *Df(2R)CA53* and $smg^{PL00423}$ (Bloomington *Drosophila* Stock Center). $smg^{PL00423}$ (BDGP Gene Disruption Project) contains a *P*-element insertion in the first intron of *smg*. This mutant was characterized by analysing the progeny of $smg^{PL00423}/smg^{1}$ and $smg^{PL00423}$/*Df(3L)scf-R6* mothers crossed with wild-type males. None of the embryos hatched (n>400) consistent with $smg^{PL00423}$ being a mutant allele of *smg*.

DNA constructs and transfection

The *Act88F-PABPN1-17ala* transgene was constructed as follows. A 2kb fragment of genomic DNA from *Act88F* [38] was amplified by PCR using primers 5'GAATGCACAATAGGCAAATTTAGTT and 5'GGCAGTTGTTTATCTGGAAGGGAG. The PCR fragment was cloned into a pGemT-easy vector (Promega), digested with *EcoRI* and cloned upstream of PABPN1-17ala present between *EcoRI* and *XbaI* sites into a CaSpeR4 vector. Several transgenic stocks were generated by *P*-element transformation using the w^{1118} stock and standard methods. Reporter constructs for HEK293T transfection were generated as follows. The mouse Ndufa10 3'UTR was amplified by PCR (Platinium Taq HF, Life technologies) using genomic DNA from FVB mice with the primers 5'CTAGTCTAGAAGGGTCCCCTCCGCCCAGCCGT and 5'TTTGCGGCCGCTGAGTGCTAAATTTCATTAA. The SRE sequence was mutated (creating a *Hpa*I restriction site) by performing three consecutive PCR. PCR1 was performed with primers 5'AGGGTCCCCTCCGCCCAGCCGT and 5'CATCACTAGAAGG**GTTAAC**AACCTTTTCAG. PCR2 was performed with primers 5'CTGAAAAGGTT**GTTAACCC**TTCTAGTGATG and 5'TTAATGAAATTTAGCACTCA. The last PCR was performed using PCR1 and PCR2 products at equimolar concentrations with primers 5'TAGTCTAGAAGGGTCCCCTCCGCCCAGCCGT and 5'TTTGCGGCCGCTGAGTGCTAAATTTCATTAA. The PCR fragments (SRE+ and SRE-) were cloned downstream of the Renilla Luciferase cDNA in the pRLTK-luciferase (gift from Dr F. Rau) between *Xba*1 and *Not*I restriction sites. Positive clones (pRLTK-Luc-SRE+ and pRLTK-Luc-SRE-) were verified by DNA sequencing. Transfection of human HEK293T cells was performed using PEI in 12-wells plate with DMEM, 10% FBS and gentamycin. The pRLTK-Luc-SRE+ and pRLTK-Luc-SRE- constructs were co-transfected with the PGK-eGFP plasmid as control for transfection. Cells were harvested 48 hours after transfection for RNA extraction.

Microarrays

The microarray procedure performed with the OPMD *Drosophila* model has been previously published [26] and the dataset is available at GEO repository under the accession number GSE64094. Genome-wide expression from adult thoraxes was compared between flies expressing PABPN1-17ala (*UAS-PABPN1-17ala/+; Mhc-Gal4/+*) and control flies (*Mhc-Gal4/+*) at three time points, using a two-way ANOVA with genotype and time as the first and second variables. Genes were considered differentially expressed when the *p*-value was < 7.1E-7 (Bonferroni corrected) [26]. The A17.1 mouse microarray dataset has been previously published [54] and is available at GEO repository under the accession number GSE26604. In this dataset, genome-wide expression profile of skeletal muscles from A17.1 mouse was compared to controls. Statistical analysis included a cut-off *p*-value of 0.05 and FDR correction [54].

Analysis of wing position

Wing position defects were scored as described previously [23].

Antibodies, western blots, immunostaining and immunoprecipitations

Antibodies used for western blots were guinea pig anti-Smg (1:5000) (gift from C. Smibert), rabbit anti-Smg (1:1000), rabbit anti-PABPN1 (1:2500) [85], mouse anti-α-Tubulin (1:20000) (T5168, Sigma), mouse anti-HA (1:25000) (Invitrogen), rabbit anti-GFP (1:4000) (Invitrogen), rabbit anti-PABP2 (1:1000) [73] and rabbit anti-human SAMD4A (1:2000) [86]. Immunostaining were performed with rabbit anti-Smg (1:2500), rabbit anti-PABPN1 (1:1000) and rabbit anti-SAMD4A (1:1000). For protein co-precipitations, Smg immunoprecipitations were performed using 50 dissected thoraxes homogenized in 20 mM Tris HCl pH 8.0, 150 mM NaCl, 0.2% NP-40, 1.5 mM DTT, 10 mM EDTA pH 8.0, 0.2 mg/mL Heparin, supplemented with protease inhibitor cocktail (Roche), and with either 100 U/ml RNasin, or 25 μg/ml RNase A. After three 10 min-centrifugations at 16 000 g at 4°C, the supernatant was incubated for 3 hours at 4°C, with either rabbit anti-Smg or rabbit normal IgG (Santa Cruz Biotechnology) (mock immunoprecipitation), covalently linked to Dynabeads Protein G. The beads were washed 5 times with 20 mM Tris HCl pH 8.0, 150 mM NaCl, 0.01% NP-40, 1 mM DTT, 1 mM EDTA pH 8.0, 10% glycerol, supplemented with protease inhibitor cocktail. The proteins were eluted in 1X LDS NuPAGE, 50mM DTT. For RNA co-precipitations, Smg immunoprecipitations were performed using 150 thoraxes homogenized in 10 mM HEPES pH7.0, 100 mM KCl, 5 mM $MgCl_2$, 0.5% NP-40, 1 mM DTT, 100 U/ml RNasin, 200 μg/ml hydrolysed yeast tRNA and protease inhibitor cocktail. After three 10 min-centrifugations at 16000 g at 4°C, the supernatant was pre-cleared with Dynabeads Protein A (Invitrogen) for 30 min at 4°C, then incubated with rabbit anti-Smg or rabbit normal IgG (Santa Cruz Biotechnology) (mock immunoprecipitation) for 3 hours at 4°C, and with Dynabeads Protein A for 1 hour at 4°C. The beads were washed 5 times with 10 mM HEPES pH 7.0, 100 mM KCl, 5 mM $MgCl_2$, 0.5% NP-40, 1 mM DTT, 100 U/ml RNasin, 200 μg/ml hydrolysed yeast tRNA and protease inhibitor cocktail. RNA was extracted using Trizol (Invitrogen), followed by DNA digestion with TURBO DNase (Ambion), and quantified by RT-qPCR using the LightCycler® 480 Instrument (Roche). *sop* was used as control mRNA.

Quantitative PCR and Poly(A) test assays

Primers used for quantitative PCR and for PAT assays were as follows.

RT-qPCR (*Drosophila*)

Gene	Forward primer	Reverse primer
CG5703	TTTTGGAAACGCCAGAATG	GCGATGGACGAACAGATTG
ND75	CTGCAAGAAGCTGAAGAAGC	TCAACAGGAACAGCACCTTG
CG12079	GCTCTGTCGGCCAAACAT	TGTACTGGCCATCCGAAGA
ND42	AAAGTCTGGCGTGATGTCTG	TCTAGATGCCCTGGTTGATG
CG15434	TTCCGATTGGCCAGTTTTAC	AGGTCGGGATTGCTCTTCTT

ND23	TGCATCTACTGCGGATTCTG	TCTCCTTGTTGTACAGCAGCTC
SdhA	GCCAATGCCCAGATGACTAT<br>CGTGTGCCATCGATTTTG	GCTTCTTCTGCTGACCATCC<br>CCGTGTGTGATGTGGTAACTG
SdhB	GAGGCGAGACAGATCCTGAG	TCCAGCGGTAGATCTCGAAC
CG10219	GCTGCCTTCATAGCTCCATC	GGACGTTTCCAACGACAGAT
Ucrh	CGTCAGAGCTGATGACGAAG	CACGATCATTGCACTCTTGG
CG14482	AACATTTTGGTGCGTGTGAA	GTAGGACCAGCTTCCAGTCG
CG3560	GCAGATGGGCCTACAATCTC	GGCAGAATGGTCTTGGTCAT
RFeSP	CTCTCCACGGGATTGAAGG	CGACTGTCCTTCACGGACTC
ox	GTCGGCCTTTTTCTTCGAG	CGTATTTGCCCTTGATGTCC
CG2249	GCACCCACTGCGAACTTTAT	TGATGCGGTACTTGTTCGTC
CG18809	AAGTGAAGACGCCCAATGAG	CAGGCGTTGCTCTTGTACTTG
CG9603	CCCAGCAAAATGATGAACCT	GTAGAGCACGTTGTCCACGA
CG7181	GTCGGTCGTCTCTGGACCT	CTTGTAGTCCCGGATGTGGT
cype	GGTGACCATCGCCTACAAAA	CCTAGCAGGACTGGAAACGA
ATPsyn-beta	TGAGGCTCCTGAATTCGTTC	CAACACAGTTTTGCCCACAC
sun	ACTGACCGGATTCAGAAAGG	CGGCGATGTTGGAGTATTG
Oscp	CCCCATCATCAACAAGAAGG	GCTTCTTTAGACGTCCGTTGTC
CG4692	ATGGCATTCGGTGACTATCC	ATCTCGCCCAGCTTGACC
CG1746	TGCCACAGATCAGGTCATTC	GTAGCCGATGATGAGGGAAC
ATPsyn-b	TGAGGCTCCTGAATTCGTTC	CAACACAGTTTTGCCCACAC
sop	CACCCCAATAAAGTTGATAGACCT	ACCACCACGAGAGCCAAAT
Cpr100A	AGGAGGATGGCATCAACTTC	GGCAAGTGATCTCCAGAAGC
spargel	AGACGTGCCTTCTGTCGTTC	AGCTTCGAAAGAGTCCTCGAC
ewg	GATCGAAATGCTGGAAGAGG	GCCACATCATCATTCACTGG
delg	CGCTTGAACAGCGGATTATG	TAGGAACTGCCATAGCTGCAC
dERR	TGCCACTTAACGACCAGATG	CACTCCTTGGCCAAATGTTC
smg	TCCCAAAATGCATCACGTC	TCGCATTTGCGAGTCTTACC
RpS6	ATATCCTCGGTGACGAGTGG	TCCCTTCTTCAGGAGCAGAC
RpL32	CTTCATCCGCCACCAGTC	CGACGCACTCTGTTGTCG
Vha16-1	CATCATTCCTGTGGTCATGG	GCTCCCAAGTGAATGAAGC
CG1031	AGCAAGACGTAGTGGTTGTCAC	TCCTTGAGACCAGCATTTCC

RT-qPCR (mouse)

Gene	Forward primer	Reverse Primer
Ndufs3	GGATCACACCAATGCACAATT	AGTTGAACCGCAGAGACAGC
Ndufv2	CAGAATGGATGGCTACCTATCTC	GATATGGTACTTCCCAACTGGC
Ndufa10	AGTATGCAGATGCCCTGGAG	GCTCCAAGACCACACCTTGTC
Cox5a	CTGTCTGTTCCATTCGCTGC	CAGATCATAGCCAACAAGTGTATTC
Uqcrc1	CCTCGCATGCTACCTGCA	CGACTGCCAGCGTCAAT
Cyp27a1	ACTCAGGAGACCATCGGCA	CCATGTCAGTGTGTTGGATGT
Atp5f1	ATCGACATGGAGAAGGCACA	GTACTCCAGGCGATTCTTTACCTC
RpL0	GAGGACCTCACTGAGATTCGG	TTCTGAGCTGGCACAGTGAC

RT-qPCR (human)

Gene	Forward primer	Reverse Primer
NDUFV1	GAGGCCTCCAATCTGCAG	GCCAATCAGACCTGCCTC
NDUFS1	CACAGCAGCAAGCAACTTGAT	ATGCCAACCTTCTCACAAGC
NDUFB11	GACCACACCGTGGCAAGA	GTCATAACCATGGGAGTCTGG
ATP5B	ATAACATCTTTCGCTTCACCCA	CATCAGCAGGCACATAGATAGC
ATP5F1	CAACAGGCACTGGTTCAGAAG	GTAACTTCCAAAGCCATAGCAATG
B2M	CTCTCTTTCTGGCCTGGAGG	TGCTGGATGACGTGAGTAAACC

RT-qPCR (following transfection in HEK293T cells)

Gene	Forward primer	Reverse primer
luciferase	AACGCGGCCTCTTCTTATTT	TTTGCCTGATTTGCCCATA
GFP	GCTACCCCGACCACATGAAG	CGGGTCTTGTAGTTGCCGT
hSAMD4A	TACGATTGGAACCAGCACAAGT	ATTCATGGGTGGGGTAAGGGA

qPCR

Gene	Forward primer	Reverse primer
mt :CoI	GGTGCTCCTGATATAGCATTCC	CTTGTTCCAGCTCCATTTTC
mt :CoII	CACCTGGACGATTAAATCAAAC	AGCCCCACAGATTTCTGAAC
mt :Cyt-b	AGACCAAATTTATTGGGAGACC	GGTTGAATATGGGCAGGTG
RpL32	CTGTGAGAGTTCGCCAAATG	CATTGAGTTTCCGGTGTGTC
MT-RNR1	ACACATGCAAGCATCCC	GGCTGGCACGAAATTGA
B2M	TTGTCTTTCAGCAAGGACTG	ATCTTGGGCTGTGACAAAGT

RT-qPCR of uncleaved RNAs

Gene	Forward primer	Reverse primer
ND42	CCTCACCAAGACGAAAATAGGC	CGATATTTACCATATTCGCTTTTTAAC
CG12079	GCTCCAGCCAAGAAGTAATCC	TAGGAACGAGCCAAGAAAGC
Ucrh	GATCATTGCGTTTCGCACAGTC	GTATTACCTACCCAATTTCGCTTC
RpS6	ACCAAATGATCCACGAGAGG	AGCTTACACTCGCTCAATCG
RpL32	TGTAACGTGGTCGGAATACAC	ACTGATATCCATCCAGATAATGCA
sop	GAACACTTTCGAGGCCAATC	CTACAACAGAATCTCCAAATCGACC
Mhc	GGGAGATAAGATCATCACTTGACAC	ATGAATGAATATGTCCCAGTCG
Act88F	GCTCCATGTCGAGTAGCAATC	GGGGCGGGTAATAGAAAAATG
Cpr100A	CTATGTTAATTTGGGCCTTGG	ACAAAAGACTGCAGGACACG

PAT/ePAT assays (*Drosophila*)

Gene	Forward primer
CG5703	ACGACCTGAAGGCGGATAAG
CG12079	TGATGAGAAGAAGCGAGTCGT
SdhB	CACAAGTAGGGCCCAAGTCC
Ucrh	GATCATTGCGTTTCGCACAG
ox	CTCGATGTCACGTCGGTTG
ND75	GGAAGAGCCAGCGCAGTAG
SdhA	CACAAGTAGGGCCCAAGTCC
RFeSP	ACGAGGGTCTTCTCGTGGTC
CG2249	TGGCGACAGCAACAACAAAT
CG4692	GGTCCTGCATCTGCAACAG
ATPsyn-beta	GGCAAAGGAAGCTGCCTAGA
sop	GGATTGCTACACCTCGGCCCGT
Act88F	CTCATCCTTCATGGCCATTTC
TpnC41C	TCCGATGGATCGGGTACTGT
smg	CGTAGACGAATGTGCATAAGTCAG

ePAT assays (mouse)

Gene	Forward primer
Ndufs3	GACGATGAGGTAAAGCGGGT
Ndufa10	GCCTGGGCAGAGATGAAAGA
Cyp27a1	GGAGAACTCTGTGCCCTTCC
RpL32	GTGCTGCTGATGTGCAACAA

Patients

Skeletal muscle biopsies from 4 control and 4 OPMD aged-matched individuals were selected for the proteomic analysis. OPMD patients showed typical clinical phenotype and the PABPN1 mutation was confirmed. All muscle biopsies were obtained during surgical procedure after informed consent in accordance with the French legislation on ethical rules.

Preparation of muscle samples for label-free LC-MS/MS analysis

Muscles specimens were freshly dissected, quick-frozen in liquid nitrogen, transported on dry ice and then stored at -80ºC prior to usage. Equal amounts of tissue were employed for the preparation of muscle extracts from normal versus pathological specimens. Skeletal muscle tissue was homogenised using a hand-held IKA T10 Basic Homogeniser (IKA-Labortechnik, Staufen, Germany). Crude extracts were then incubated for 2.5 hours at room temperature with agitation using a Thermomixer from Eppendorf (Hamburg, Germany). Samples were centrifuged at 4°C for 20 min at 14,000*g* and the urea-soluble protein containing middle layer was retained for further analysis. For comparative proteomic profiling, skeletal muscle homogenates from normal (n=4) and pathological (n=4) samples were pre-treated with the Ready Prep 2-D clean up kit from BioRad Laboratories (Hemel-Hempstead, Hertfordshire, UK). The resulting protein pellets were resuspended in label-free solubilisation buffer, consisting of 10 mM Tris, pH 8.0, 6 M urea and 2 M thiourea in LC-MS grade water. Following vortexing, sonication and centrifugation, the protein concentration of the suspensions was determined. Volumes of protein suspensions were equalized using label-free solubilisation buffer and then reduced for 30 minutes with 10 mM DTT and alkylated for 20 minutes in the dark with 25 mM iodoacetamide in 50 mM ammonium bicarbonate. The proteolytic digestion of proteins was carried out in 2 steps. Firstly, digestion was performed with sequencing grade Lys-C at a ratio of 1:100 (protease/protein) for 4 hours at 37 ºC, followed by a dilution with 4 times the initial sample volume in 50 mM ammonium bicarbonate. Secondly, further digestion was based on incubation with sequencing grade trypsin at a ratio of 1:25 (protease/protein) overnight at 37ºC. The protease-treated muscle protein suspensions were diluted 3:1 (v/v) with 2% trifluoroacetic acid in 20% acetonitrile. To ensure an even suspension of peptide populations from WR versus WT muscle, the samples were briefly vortexed and sonicated.

Label-free LC-MS/MS analysis

The nano LC-MS/MS analysis of normal versus pathological samples was carried out with the help of an Ultimate 3000 nanoLC system (Dionex) coupled to an LTQ Orbitrap XL mass spectrometrer (Thermo Fisher Scientific, Dublin, Ireland). Data was acquired with Xcalibur software, version 2.0.7 (Thermo Fisher Scientific). The MS apparatus was operated in data-dependent mode and externally calibrated. Survey MS scans were acquired in the Orbitrap in the 300-2000 *m/z* range with the resolution set to a value of 30 000 at *m/z* 400 and lock mass set to 445.120025 u. CID fragmentation was carried out in the linear ion trap with the three most intense ions per scan [87].

Quantitative profiling by label-free LC-MS/MS analysis

Processing of the raw data generated from LC-MS/MS analysis was carried out with Progenesis label-free LC-MS software (version 3.1; Non-Linear Dynamics, Newcastle upon Tyne, UK). Prior to exporting the MS/MS output files to MASCOT (www.matrixscience.com) for protein identification, a number of criteria were employed to filter the data including: (i) peptide features with ANOVA < 0.05 between experimental groups, (ii) mass peaks (features) with charge states from +2, +3, and (iii) greater than one isotope per peptide. A MASCOT generic file was generated from all exported MS/MS spectra from Progenesis software. The MASCOT generic file was used for peptide identification with MASCOT (version 2.2) and searched against the UniProtKB-SwissProt database. Importantly, the following criteria were applied to assign a skeletal muscle-

associated protein as properly identified: (i) an ANOVA score between experimental groups of ≤0.05, (ii) proteins with ≥1 peptides matched and (iii) a MASCOT score >40.

Supporting References

74. Schuster CM, Davis GW, Fetter RD, Goodman CS (1996) Genetic dissection of structural and functional components of synaptic plasticity. I. Fasciclin II controls synaptic stabilization and growth. Neuron 17: 641-654.
75. Dahanukar A, Walker JA, Wharton RP (1999) Smaug, a novel RNA-binding protein that operates a translational switch in Drosophila. Mol Cell 4: 209-218.
76. Sigrist SJ, Thiel PR, Reiff DF, Lachance PE, Lasko P, et al. (2000) Postsynaptic translation affects the efficacy and morphology of neuromuscular junctions. Nature 405: 1062-1065.
77. Zaffran S, Astier M, Gratecos D, Semeriva M (1997) The held out wings (how) Drosophila gene encodes a putative RNA-binding protein involved in the control of muscular and cardiac activity. Development 124: 2087-2098.
78. Markesich DC, Gajewski KM, Nazimiec ME, Beckingham K (2000) bicaudal encodes the Drosophila beta NAC homolog, a component of the ribosomal translational machinery*. Development 127: 559-572.
79. Mahone M, Saffman EE, Lasko PF (1995) Localized Bicaudal-C RNA encodes a protein containing a KH domain, the RNA binding motif of FMR1. The EMBO journal 14: 2043-2055.
80. Lantz V, Chang JS, Horabin JI, Bopp D, Schedl P (1994) The Drosophila *orb* RNA-biding protein is required for the formation of egg chamber and establishment of polarity. Genes and Development 8: 598-613.
81. Christerson LB, McKearin DM (1994) orb is required for anteroposterior and dorsoventral patterning during Drosophila oogenesis. Genes & development 8: 614-628.
82. Murata Y, Wharton RP (1995) Binding of pumilio to maternal hunchback mRNA is required for posterior patterning in Drosophila embryos. Cell 80: 747-756.
83. Barker DD, Wang C, Moore J, Dickinson LK, Lehmann R (1992) Pumilio is essential for function but not for distribution of the Drosophila abdominal determinant Nanos. Genes & development 6: 2312-2326.
84. Lin H, Spradling AC (1997) A novel group of pumilio mutations affects the asymmetric division of germline stem cells in the Drosophila ovary. Development 124: 2463-2476.
85. Krause S, Fakan S, Weis K, Wahle E (1994) Immunodetection of poly(A) binbing protein II in the cell nucleus. Exp Cell Res 214: 75-82.
86. Baez MV, Boccaccio GL (2005) Mammalian Smaug is a translational repressor that forms cytoplasmic foci similar to stress granules. The Journal of biological chemistry 280: 43131-43140.
87. Jockusch H, Holland A, Staunton L, Schmitt-John T, Heimann P, et al. (2014) Pathoproteomics of testicular tissue deficient in the GARP component VPS54: the wobbler mouse model of globozoospermia. Proteomics 14: 839-852.
