# Supplementary figures and images for: Mitochondrial Dysfunction Reveals the Role of mRNA Poly(A) Tail Regulation in Oculopharyngeal Muscular Dystrophy Pathogenesis
Source: PLoS Genet. 2015 Mar 27;11(3):e1005092. doi: 10.1371/journal.pgen.1005092 (PMC4376527; doi:10.1371/journal.pgen.1005092)

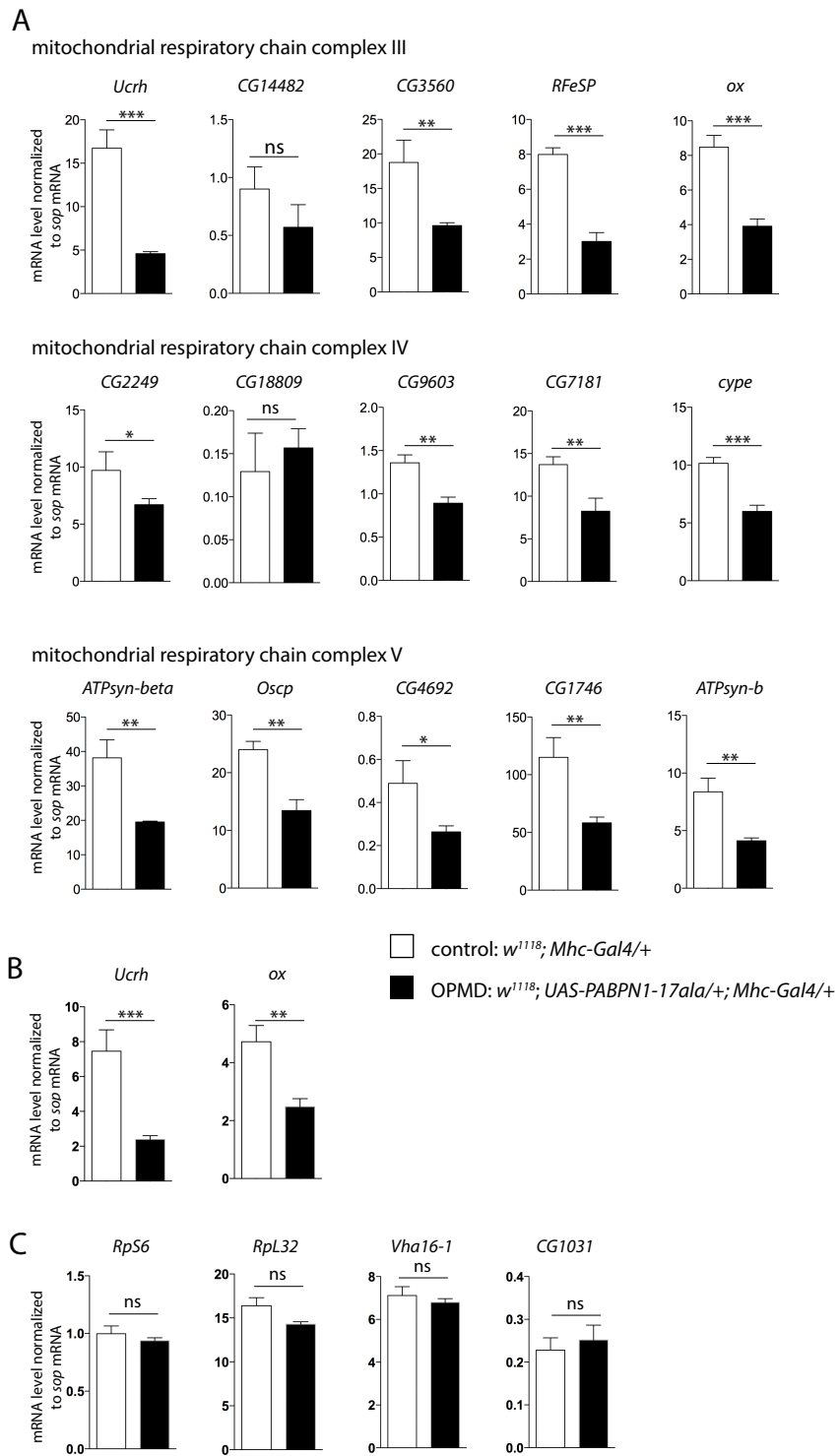

Figure S1

Supplement: S1 Fig — A) Quantification of mRNA levels in control and PABPN1-17ala-expressing adult thoraxes at day 2, using RT-qPCR. mRNA levels were normalized to sop mRNA. Means are from three biological replicates, error bars represent standard deviation. * p-value <0.05, ** p-value <0.01, *** p-value <0.001, ns: not significant, using the Student’s t-Test. Genotypes are indicated for (A-C). B) Quantification of mRNA levels at day 2, using RT-qPCR, with reverse transcription performed with random hexamers, showing down-regulation in PABPN1-17ala-expressing thoraxes compared to control. mRNA levels were normalized to sop mRNA. Means are from two biological replicates quantified three times, error bars represent standard deviation. ** p-value <0.01, *** p-value <0.001, using the Student’s t-Test. C) Quantification of negative control mRNAs which were found unaffected in microarray analysis, using RT-qPCR. mRNA levels were normalized to sop mRNA. Means are from two biological replicates quantified three times, error bars represent standard deviation. ns: not significant, using the Student’s t-Test. (PDF) [file pgen.1005092.s002.pdf]

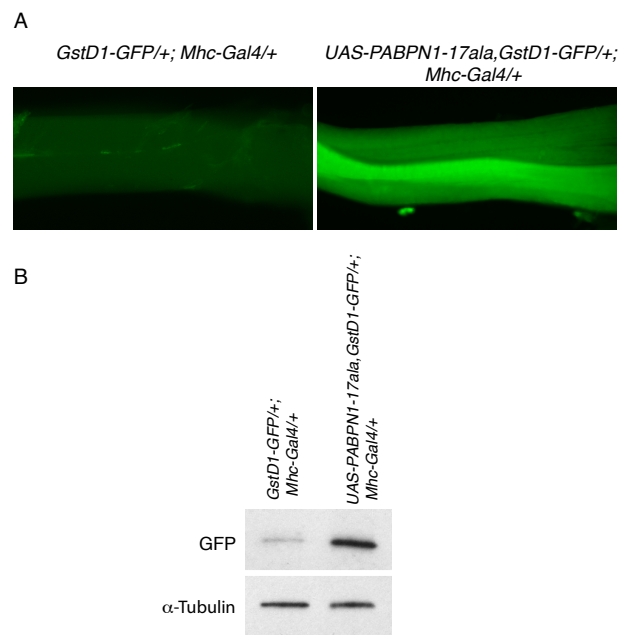

Figure S2

Supplement: S2 Fig — A) GFP expression in indirect flight muscles of control (GstD1-GFP/+; Mhc-Gal4/+) and OPMD (UAS-PABPN1-17ala, GstD1-GFP/+; Mhc-Gal4/+) flies at day 2. Expression of the GstD1-GFP transgene was visualized by direct fluorescence and captured with the same settings for both genotypes. B) Western blots of control (GstD1-GFP/+; Mhc-Gal4/+) and OPMD (UAS-PABPN1-17ala, GstD1-GFP/+; Mhc-Gal4/+) adult thoraxes at day 6 to quantify GstD1-GFP protein. Protein extracts were from 0.25 thoraxes of flies raised at 18°C. α-Tubulin was used as loading control. (PDF) [file pgen.1005092.s003.pdf]

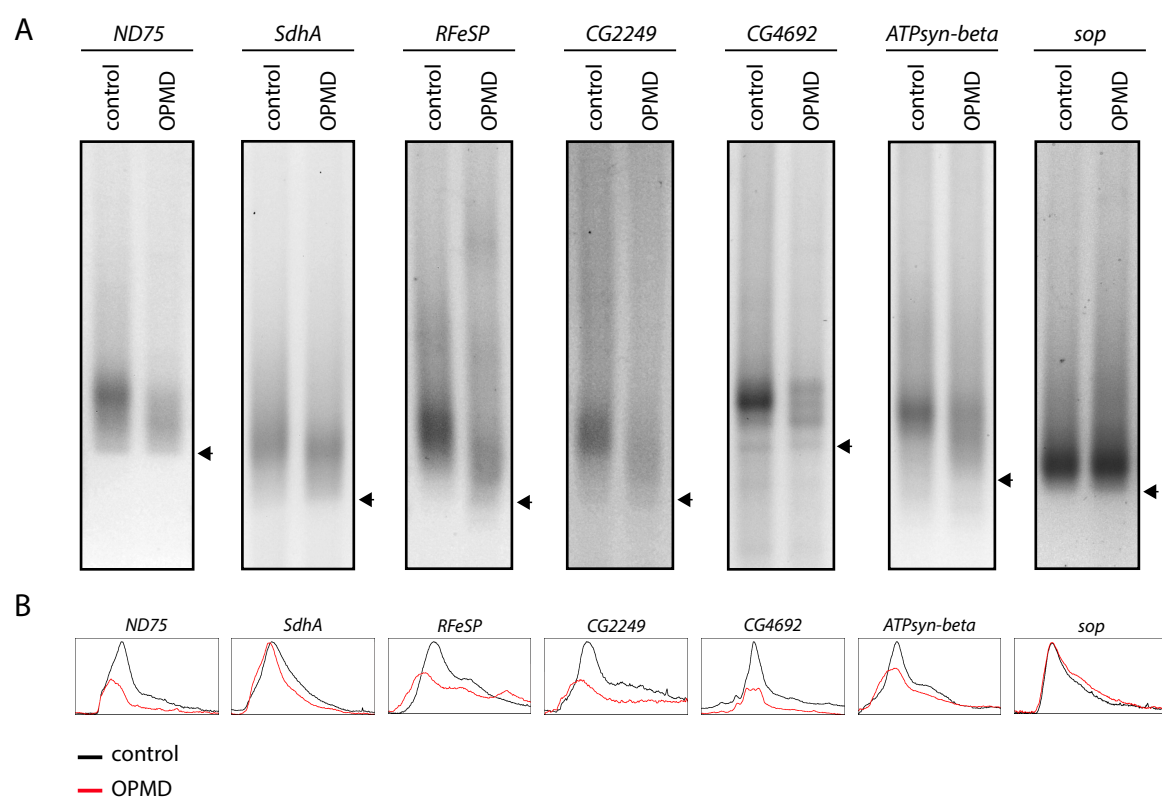

Figure S3

Supplement: S3 Fig — A) ePAT (extension PAT) assays of mRNAs encoding mitochondrial proteins in control (w 1118) and OPMD (Act88F-PABPN1-17ala/+) adult thoraxes at day 6. sop mRNA was used as a negative control. Arrows indicate poly(A) tails of 12A. B) Profiles of ePAT assays shown in A, using ImageJ software. (PDF) [file pgen.1005092.s004.pdf]

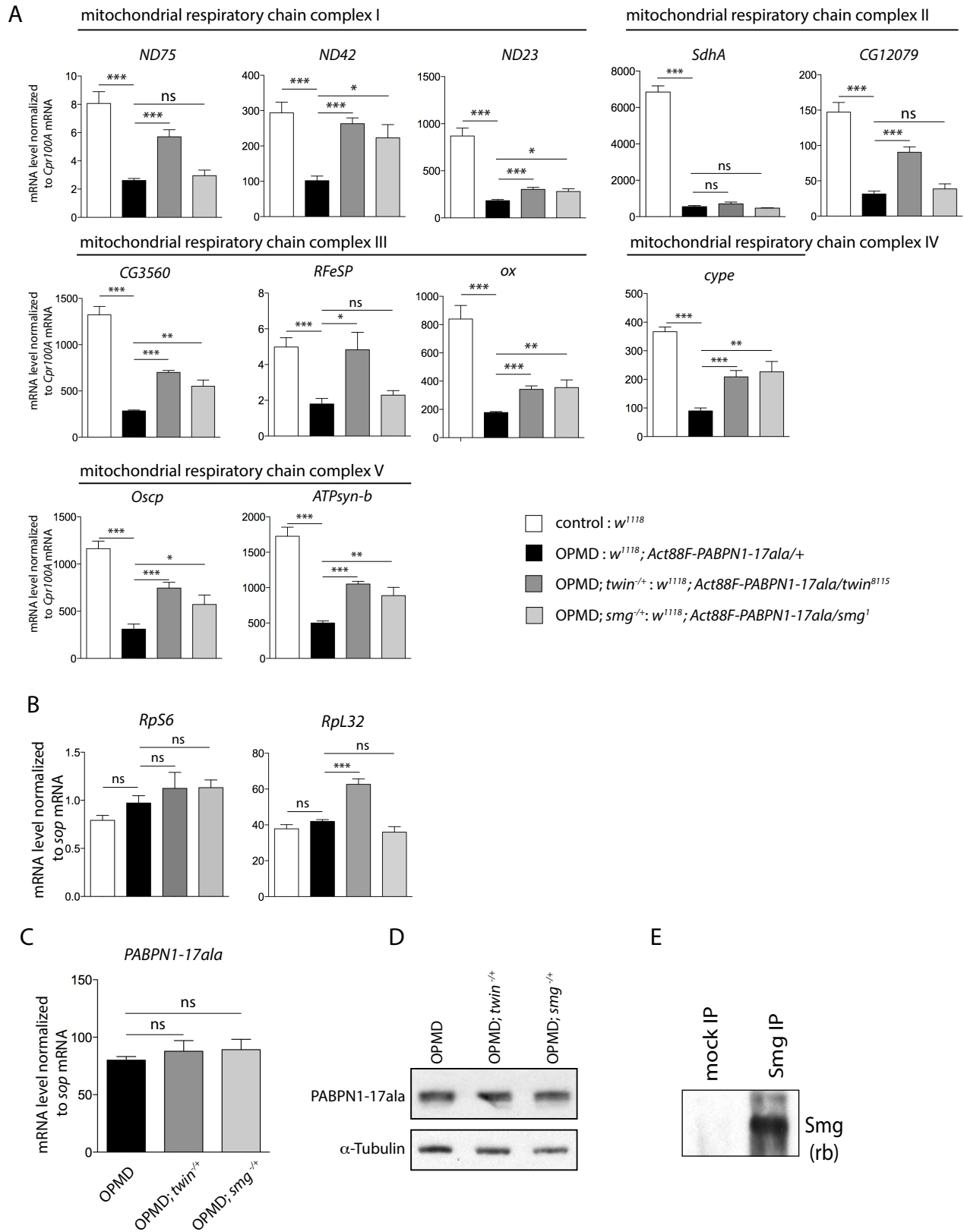

Figure S4

Supplement: S4 Fig — A) Quantification of mRNA levels in control and PABPN1-17ala-expressing thoraxes in the presence or absence of heterozygous twin or smg mutations at day 6, using RT-qPCR. mRNA levels were normalized to Cpr100A mRNA. Means of two biological replicates quantified three times. Error bars represent standard deviation. * p-value <0. 05, ** p-value <0.01, *** p-value <0.001, ns: not significant, using the Student’s t-Test. Genotypes are indicated for (A-D). B) Quantification of negative control mRNAs which were unaffected in PABPN1-17ala-expressing muscles in the microarray analysis, using RT-qPCR. mRNA levels were normalized to sop mRNA. RpS6 mRNA levels remained unaffected in the different genotypes. The upregulation of RpL32 mRNA in the twin -/+ condition can be explained by its reduced deadenylation, since the CCR4-NOT complex can act without specificity. Means are from two biological replicates quantified three times, error bars represent standard deviation. *** p-value <0.001, ns: not significant, using the Student’s t-Test. C) Quantification of PABPN1-17ala mRNA in thoraxes at day 6 using RT-qPCR, either in the absence (w 1118 ; Act88F-PABPN1-17ala/+) or the presence of twin (w 1118 ; Act88F-PABPN1-17ala/twin 8115) or smg (w 1118 ; Act88F-PABPN1-17ala/smg 1) mutations. mRNA levels were normalized to sop mRNA. Mean quantifications are from two biological replicates quantified three times. Error bars represent standard deviation. ns: not significant using the Student’s t-Test. D) PABPN1-17ala protein levels determined by western blots. Protein extracts were from 0.25 thoraxes at day 6. α-Tubulin was used as a loading control. E) Western blot validating Smg immunoprecipitation from thoraxes in the conditions used for RNA co-precipitations shown in Fig. 5E. The western blot was revealed with rabbit (rb) anti-Smg antibody. (PDF) [file pgen.1005092.s005.pdf]

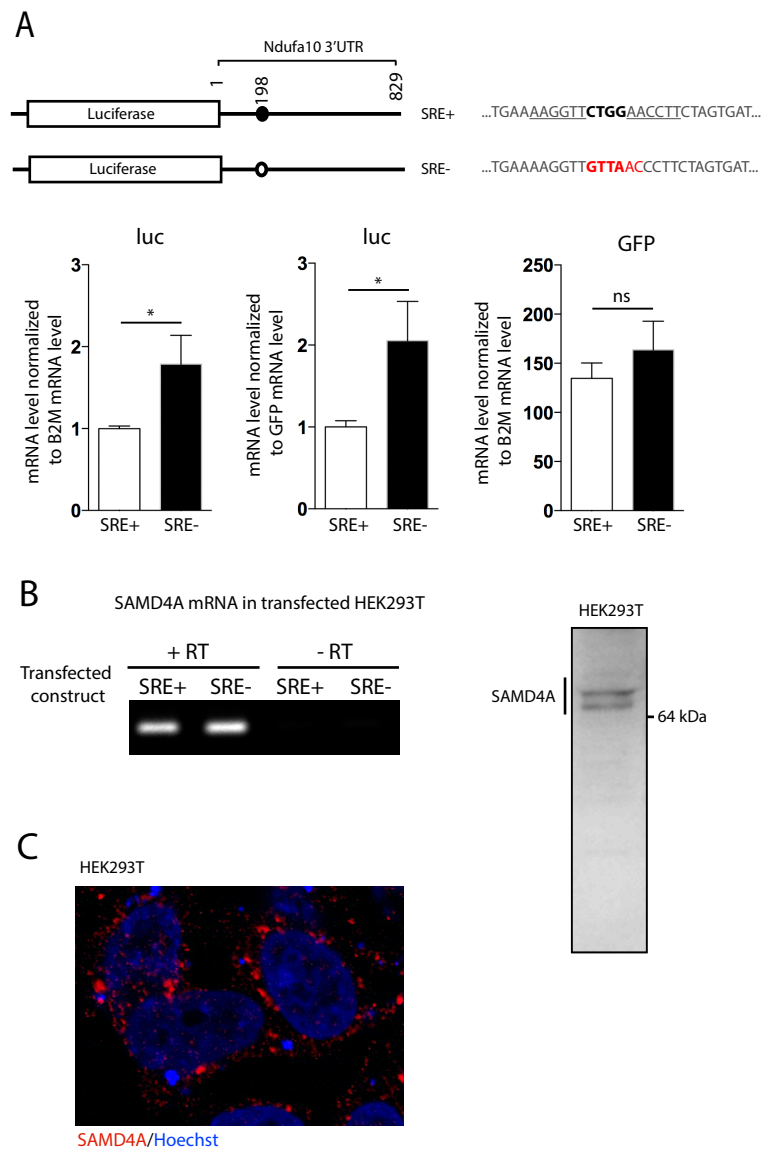

Figure S5

Supplement: S5 Fig — A) Quantification of mRNAs produced from SRE+ and SRE- reporter constructs transfected in HEK293T cells. (Top) Schematic representation of the 3'UTR of the Ndufa10 gene containing the SRE sequence (black circle, SRE+) or mutated in the SRE (open circle, SRE-), downstream of the Renilla Luciferase gene. Sequences of the SRE region are shown. Nucleotides in the SRE loop (CTGG) are in bold, those in the stem are underlined. The SRE mutation (SRE-) was designed to create a Hpa1 restriction site (red). (Bottom) HEK293T cells were co-transfected with either pRLTK-Luc-SRE+ or pRLTK-Luc-SRE-, and PGK-eGFP constructs. mRNA levels were quantified using RT-qPCR, normalized to B2M or GFP mRNA levels. The levels of GFP mRNA normalized to B2M mRNA were similar in both types of transfection (right panel). Means are from eight quantifications performed in three independent transfections, error bars represent standard deviation. * p-value <0.05, using the Student’s t-Test. B) RT-PCR (left) and western blot (right) showing that HEK293T cells express SAMD4A at the mRNA and protein levels. For RT-PCR, negative controls in which the reverse transcriptase was omitted (-RT) are shown. The western blot was revealed with anti-human SAMD4A. C) Confocal images of SAMD4A immunostaining in HEK293T cells with anti-human SAMD4A (red) showing accumulation of SAMD4A in foci. DNA was revealed with Hoechst (blue). (PDF) [file pgen.1005092.s006.pdf]

A

1) Poly(A) tail digestion

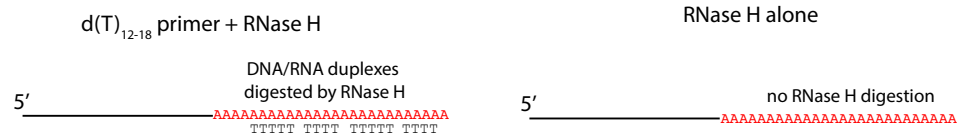

2) ePAT

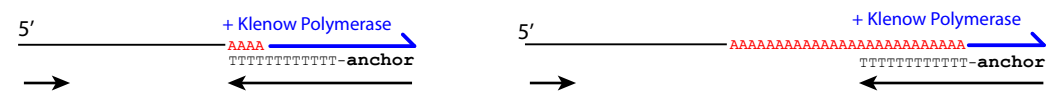

B

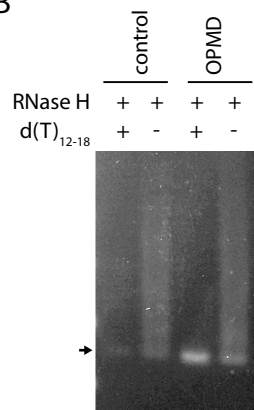

C

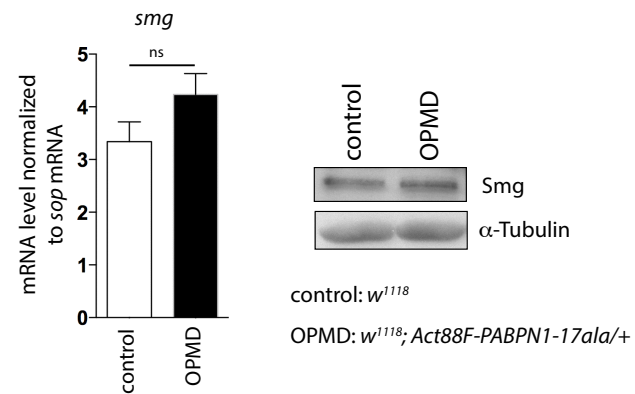

Figure S6

Supplement: S6 Fig — A-B) Poly(A) site utilization in control and PABPN1-17ala-expressing muscles. A) Scheme of the experiment. RNAs were treated with RNase H either in the presence or the absence of oligo-d(T)12-18 to degrade the poly(A) tail (1), and then subjected to ePAT (2). Black arrows indicate the primers used in the ePAT reaction. The oligo(A) remaining of the poly(A) tail after RNase H digestion allows to produce a discrete band in PCR amplification which indicates the site used for polyadenylation. B) Results of the experiment described in (A). The same poly(A) site was used in both control and PABPN1-17ala-expressing muscles (arrow). Genotypes are indicated in (C). C) Quantification of smg mRNA and protein levels in control and PABPN1-17ala expressing muscles at day 6. Quantification of smg mRNA using RT-qPCR. mRNA levels were normalized to sop mRNA (left). Means are from two biological replicates quantified three times, error bars represent standard deviation. ns: not significant, using the Student’s t-Test. Western blot of control and PABPN1-17ala-expressing thoraxes revealed with rabbit anti-Smg (right). Protein extracts were from 5 thoraxes. α-Tubulin was used as a loading control. (PDF) [file pgen.1005092.s007.pdf]
